# The role of booster vaccination in decreasing COVID-19 age-adjusted case fatality rate: Evidence from 32 countries

**DOI:** 10.3389/fpubh.2023.1150095

**Published:** 2023-04-18

**Authors:** Cui Zhou, Åsa M. Wheelock, Chutian Zhang, Jian Ma, Kaixing Dong, Jingxiang Pan, Zhichao Li, Wannian Liang, Jing Gao, Lei Xu

**Affiliations:** ^1^Vanke School of Public Health, Tsinghua University, Beijing, China; ^2^Institute for Healthy China, Tsinghua University, Beijing, China; ^3^Respiratory Medicine Unit, Department of Medicine, Centre for Molecular Medicine, Karolinska Institutet, Stockholm, Sweden; ^4^Key Laboratory of Land Surface Pattern and Simulation, Institute of Geographic Sciences and Natural Resources Research, Chinese Academy of Sciences, Beijing, China; ^5^Department of Respiratory Medicine, University of Helsinki, Helsinki, Finland

**Keywords:** COVID-19, vaccination, age-adjusted case fatality rate, XGBoost (Extreme Gradient Boosting), SHapley Additive exPlanations

## Abstract

**Background:**

The global COVID-19 pandemic is still ongoing, and cross-country and cross-period variation in COVID-19 age-adjusted case fatality rates (CFRs) has not been clarified. Here, we aimed to identify the country-specific effects of booster vaccination and other features that may affect heterogeneity in age-adjusted CFRs with a worldwide scope, and to predict the benefit of increasing booster vaccination rate on future CFR.

**Method:**

Cross-temporal and cross-country variations in CFR were identified in 32 countries using the latest available database, with multi-feature (vaccination coverage, demographic characteristics, disease burden, behavioral risks, environmental risks, health services and trust) using Extreme Gradient Boosting (XGBoost) algorithm and SHapley Additive exPlanations (SHAP). After that, country-specific risk features that affect age-adjusted CFRs were identified. The benefit of booster on age-adjusted CFR was simulated by increasing booster vaccination by 1–30% in each country.

**Results:**

Overall COVID-19 age-adjusted CFRs across 32 countries ranged from 110 deaths per 100,000 cases to 5,112 deaths per 100,000 cases from February 4, 2020 to Jan 31, 2022, which were divided into countries with age-adjusted CFRs higher than the crude CFRs and countries with age-adjusted CFRs lower than the crude CFRs (*n* = 9 and *n* = 23) when compared with the crude CFR. The effect of booster vaccination on age-adjusted CFRs becomes more important from Alpha to Omicron period (importance scores: 0.03–0.23). The Omicron period model showed that the key risk factors for countries with higher age-adjusted CFR than crude CFR are low GDP *per capita* and low booster vaccination rates, while the key risk factors for countries with higher age-adjusted CFR than crude CFR were high dietary risks and low physical activity. Increasing booster vaccination rates by 7% would reduce CFRs in all countries with age-adjusted CFRs higher than the crude CFRs.

**Conclusion:**

Booster vaccination still plays an important role in reducing age-adjusted CFRs, while there are multidimensional concurrent risk factors and precise joint intervention strategies and preparations based on country-specific risks are also essential.

## Introduction

The COVID-19 pandemic has triggered a public health and economic crisis the like of which has not been seen for generations (1, 2). With the gradual reduction of COVID-19 restriction policies, the long-term epidemiological trend of COVID-19 is unpredictable. The risk of death from COVID-19 varies between countries, and case fatality rate (CFR) is an important indicator used to assess it. It is widely considered that the COVID-19 CFRs are affected by multidimensional factors, such as the SARS-CoV-2 variant infected (3), vaccination coverage (4), population age structure (5, 6), health service (7), disease burden (8, 9), environment (10), and so on (11). Of these, as the COVID-19 CFR is strongly associated with age, considering age structure when comparing CFR differences across countries is more helpful in minimizing potential bias (6). To best of our knowledge, studies have been conducted using age-adjusted CFRs for comparison across at most seven countries to illustrate the significant effect of confounding by the age distribution of the cases when using crude CFRs for country comparisons, however, cross-country and cross-period differences in risk factors for age-adjusted CFR have not been identified (12, 13). Therefore, it is essential to adjust COVID-19 CFRs according to patient age structure in the widest possible number of countries and to compare and clarify possible risk factors for age-adjusted CFRs with a global perspective, which could provide an updated and practical reference for future pandemic control.

Since December 2021, the global COVID-19 vaccination program has been in place and, due to declining antibody levels, booster doses of COVID-19 vaccine have subsequently been offered to eligible individuals (14). However, the limited and unbalanced medical resources result in the global inequity of both vaccination rate and further recovery rate. As of January 2023, more than 60% of the population worldwide has received at least one dose of COVID-19 vaccine, while in low-income countries, only 26% have received at least one dose (15), which possibly leads to an unbalanced protection ability and heavy burden in the overall health systems. Furthermore, the dominant strains in each period of the COVID-19 pandemic have different characteristics. For example, relative to the original variant, the Alpha strain has around 43–90% greater transmissibility along with a 42–82% higher risk of death (16), and the Delta strain has a transmission rate still faster (17), with concomitant greater risk of hospitalization and death (18, 19). The emergence of the Omicron variant brought the COVID-19 epidemic into a new pattern (20). Omicron’s immune escape properties make it more contagious than previous strains, but it appears to also be milder, usually causing less severe disease (21). Thus, analysis of risk factors for COVID-19 CFR based on international inequalities in boosters and the complexity of SARS-CoV-2 variants has greater research implications.

The complex factors involved in real-world health emergencies are more effectively analyzed with fast-evolving machine learning algorithms, such as Extreme Gradient Boosting (XGBoost) algorithm. XGBoost is a decision tree-based gradient boosting ensemble machine learning algorithm with improved performance based on other tree-based models such as Random Forest and Gradient Boosting Decision Tree (GBDT), which is well suited for solving classification and regression problems (22). It features several advantages that allow it to be effectively adapted to real-world studies: (1) the objective function can be customized and we choose the most appropriate loss function based on the distribution of the outcome variables; (2) it handles missing data by assigning it to a default direction and finding the best imputation value, which means it is more suitable for dealing with real-world data with limited matching; (3) it penalizes more complex models by LASSO and Ridge regularization to improve the generalization of the model; (4) it can detect and learn from non-linear data patterns, making it easier to identify the non-linear effects of features (23). SHapley Additive exPlanations (SHAP) is a well-established method for interpreting machine learning models (24). On the one hand, SHAP values can clarify the importance of each feature in the model, and on the other hand, SHAP values can break down a prediction to show how each feature affects the prediction. XGboost algorithm with SHAP explanation allows us to identify what factors are driving each country’s risk and enabled countries to directly address those risk factors with targeted interventions (25).

Here, we analyze COVID-19 age-adjusted CFRs across countries using the latest available database, as well as crude CFRs. The main aim is to identify the effects of vaccination coverage (e.g., booster vaccination) and other features in six dimensions that may affect heterogeneity in age-adjusted CFRs, including demographic characteristics, disease burden, behavioral risk factors, environmental risk factors, health services and trust levels, using machine learning approaches. Then, to further identify country-specific risk features that affect age-adjusted CFRs. Finally, we predicted the reduction in CFR by country with increased vaccination rates to assess the future health benefits of vaccination in each country.

## Materials and methods

### Data collection

#### COVID-19 crude CFR and age-adjusted CFR

Global daily confirmed infections and deaths of COVID-19 by age over the period of 4 February 2020 to 2 February 2022 (the latest database update time) were extracted from the COVerAGE-DB database, which contains the widest range of COVID-19 case and death data by age group for countries worldwide (26). The COVerAGE-DB database contains data for 108 countries, and after we filtered the countries for which both case and death data are available and the countries for which time series containing four time periods are available, there are 32 countries throughout the original, Alpha, Delta and Omicron periods, including Argentina, Australia, Austria, Belgium, Bulgaria, Chile, Colombia, Czechia, Denmark, Finland, France, Germany, Greece, Indonesia, Isreal, Italy, Jamaica, Japan, New Zealand, Nigeria, Peru, Philippines, Portugal, Slovakia, Slovenia, Somalia, South Korea, Spain, Sweden, Switzerland, Togo, and United State America.

Weekly crude CFRs were calculated from the number of new deaths and new cases per week. Weekly age adjusted CFRs were calculated by the direct method (13). The population structure for each country was calculated using World Bank population data for 2020, and the WHO World Standard Population was used as the standard population structure (27). In addition, before building the models, the outliers in CFRs were removed based on the interquartile range.

#### Vaccination data

Daily vaccination data from January 28, 2020 to January 31, 2022 in 32 countries were extracted from Our World in Data (OWID) and pre-processed by linear interpolation (28). The OWID COVID-19 vaccination dataset is the largest publicly aggregated global dataset on administered vaccinations by country and up-to-date in real time. The advantage of this database is that it differentiates the number of vaccine shots, including share of the population completed the initial vaccination protocol (2 doses for most vaccines, 1 or 3 for a few manufacturers) and share of the population receiving booster doses (doses administered beyond those prescribed by the original vaccination protocol - for example, a third dose of Pfizer/BioNTech vaccine, or a second dose of Johnson & Johnson vaccine) (28). Furthermore, considering that the protection offered by the COVID-19 vaccine drops sharply after 6 months (29–31), we calculated four categories of vaccination status: (1) the proportion of the population having completed the initial vaccination protocol within 6 months (fully vaccinated), (2) the proportion of the population having received a booster within 6 months (booster given), (3) the cumulative proportion of the population having completed the initial vaccination protocol, (4) the cumulative proportion of the population having received a booster.

#### SARS-CoV-2 lineage data

**Figure 1:**
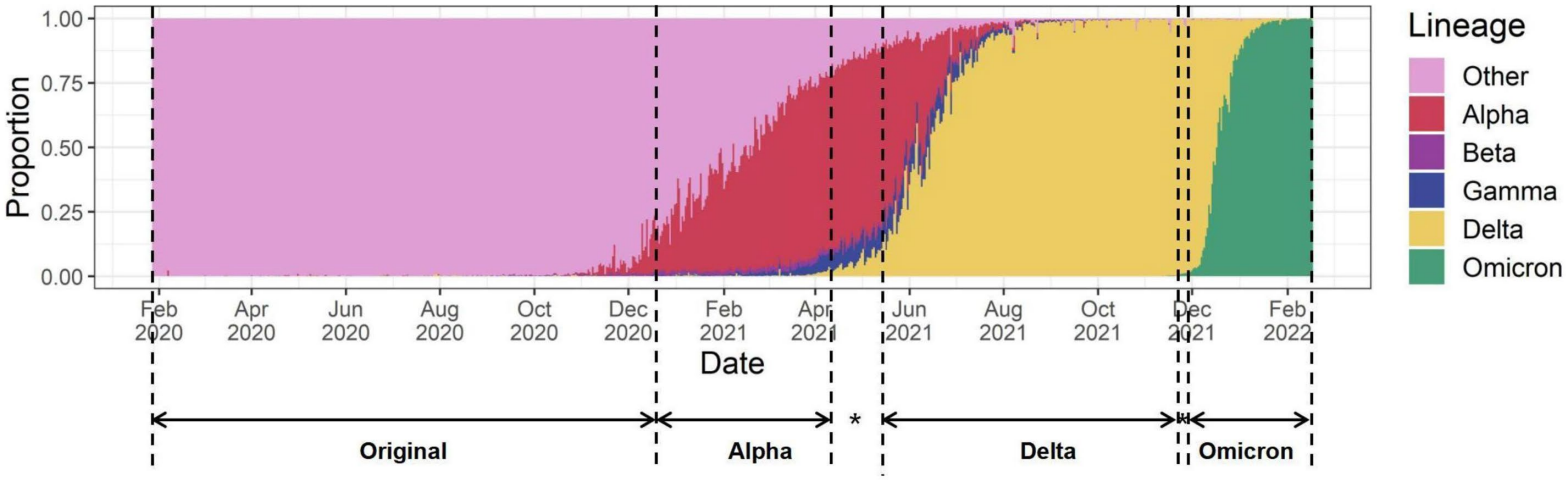
The four periods of the pandemic defined by the dominance of the different VOC strains. * A period of multiple variants mixing, with the next VOC already occurring in more than 10% of countries.

SARS-CoV-2 lineage data were obtained from the China National Center for Bioinformation (CNCB), which integrated global SARS-CoV-2 sequences from the Global Initiative on Sharing All Influenza Data (GISAID), NCBI GenBank, National Genomics Data Center (NGDC), National Microbiology Data Center (NMDC), and China National GeneBank (CNGB) and identified variants among those sequences (32). We identified those variant types that accounted for >70% of all detected sequences on a global scale for each day in the study period, and considered variants meeting that criterion as having been world-dominating. We defined periods of main VOCs dominance. The starting time of a VOC is based on the World Health Organisation’s definition of the start time of each VOC (33). The ending time was set as the next VOC occurring in no more than 10% of the countries. The COVID-19 pandemic was thus divided into four periods: the ancestral variant dominance period (original period, 28 January to 17 December 2020), Alpha variant dominance period (Alpha period, 18 December 2020 to 6 April 2021), Delta variant dominance period (Delta period, 11 May to 21 November 2021), and Omicron variant dominance period (Omicron period, 26 November 2021 to 31 January 2022; Figure 1).

#### Multi-dimensional explanatory variables

We included six dimensions of features including demographic characteristics, disease burden, behavioral risk factors, environmental risk factors, level of national health services and level of trust to comprehensively assess risk factors for COVID-19 age-adjusted CFR. Demographic characteristics include gender ratio (34), average years of schooling (35), and GDP *per capita* (36). Disease burden include the top three causes of death globally: cardiovascular diseases (CVD), stroke, and chronic obstructive pulmonary disease (COPD); comorbidity which are known to affect the outcome of COVID-19: cancers, diabetes, chronic kidney disease (CKD), and hypertension; upper and lower respiratory infections (URI and LRI), and tuberculosis (TB), which affect lung function; as well as mental disorders, acquired immunodeficiency syndrome (37), and the overall prevalence of noncommunicable diseases (NCD) (38). Behavioral risk factors include overweight (39), low physical activity, smoking, and dietary risks (37). Environmental risk factors include PM2.5 pollution (37), tree density (40), average temperature (41), and population density (42). The level of health services is indicated by the healthcare access and quality (HAQ) index (43), International Health Regulations (IHR) core capacity scores (44), health expenditure (45), number of hospitals (46), and hospital beds *per capita* (47). The trust indices include the level of people’ trust in the national government, media, and science during the pandemic (48). Data sources and detailed descriptions are detailed in Supplementary Table 1.

### XGBoost model

#### Model building

We used XGBoost to capture the non-linear associations between COVID-19 age-adjusted CFRs and vaccination rates as well as multiple dimensional features to build explanatory and predictive models. XGBoost is a decision-tree-based ensemble machine learning algorithm that uses a gradient boosting framework (23). It produces a robust, more accurate prediction model in the form of an ensemble of weak prediction models and introduces a penalty term for model complexity to provide better performance. The objective function of the XGBoost algorithm is as follows:


$$Obj\left(\theta \right)=L\left(\theta \right)+\Omega \left(\theta \right)=\underset{i}{\sum }L\left({\hat{y}}_{i},y_{i}\right)+\underset{k}{\sum }\Omega \left(f_{k}\right),f_{k}\in F$$

Where L is the training loss function. $L\left({\hat{y}}_{i},y_{i}\right)$ corresponds to the training loss function for each sample, where $y_{i}$ denotes the true value of the i sample and ${\hat{y}}_{i}$ denotes the estimated value of the i sample. Ω is regularization function that measures the complexity of the model, where k is the number of trees, F is the set of all possible regression trees.

#### Feature selection

We used a recursive feature elimination (RFE) algorithm to filter main features with the aim of retaining as few features as possible while still capturing the variation in age-adjusted CFRs (49). The RFE strategy uses all features to train a supervised model, then evaluates the features according to their importance in the model. In each iteration, only one feature with minimal model importance is eliminated, and the model fit in each iteration is compared by RMSE; ultimately, features from the better-fitting model are selected.

#### Hyperparameter tuning

The best combination of hyperparameter values was selected using a fivefold cross-validation grid search. The tuned parameters consisted of learning rate (from 0.05 to 0.2 with an interval of 0.05) and the maximum depth of the tree (from 4 to 10 with an interval of 1). The objective function was set as “reg:tweedie,” as our dependent variable of interest was zero-inflated right-skewed data. The training process was stopped when the performance of the validation dataset did not improve after further training iterations. The dataset was split into three parts: 60% for training, 20% for validation, and 20% for testing. The accuracy of the model was evaluated in terms of R^2^ and RMSE.

#### Model interpretation

We adopted the SHAP framework to rank features according to their importance and explain how booster vaccination and other key features affect the age-adjusted CFR. SHAP is a game theoretic approach that can explain the output of the XGBoost model. It connects the optimal credit allocation with a local explanation using the classical Shapley values from game theory and their associated extensions (24). The variability of the predictions is assigned to the available features, allowing evaluation of the contribution of each feature to each prediction point. SHAP provides valuable insights into a model’s behavior by overcoming the main drawback of inconsistency in classical global feature importance measures, minimizes the possibility of underestimating the importance of a feature with a certain attribution value, shows consistency and accuracy in its importance ordering, and interpreting the model’s global behavior while retaining local faithfulness. The overall importance of a feature was scored as the mean absolute value of all SHAP values for that feature, and we considered features scoring 0.1 or higher as important. The relationship between age-adjusted CFR and each key feature was examined *via* partial dependence plots, with adjustment for all other confoundings.

#### Prediction

We predicted the change in CFR for scenarios when booster vaccination was increased by 1–30% in each country. The approach is to determine the model parameters from the training and validation sets and then predict the CFRs when booster vaccination rates increase by 1–30% for each country respectively, holding all other variables constant. The principle of increasing booster vaccination is based on the actual full and booster vaccination rate in each country, so we predicted the CFRs of increasing booster vaccination rates within the range of a country’s booster vaccination rate not exceeding the cumulative proportion of the population fully vaccinated.

### Statistical analysis

Continuous data are presented as a mean with standard deviation (SD) where normally distributed and as a median with the 25th and 75th percentiles where non-normally distributed. Univariate analyses relating CFRs and multi-dimensional explanatory variables were assessed with Spearman’s rank correlation.

Analyses were performed in the R 4.1.1 and Python 3.8 environments.

## Results

### Temporal and regional heterogeneity of age-adjusted CFRs

**Figure 2:**
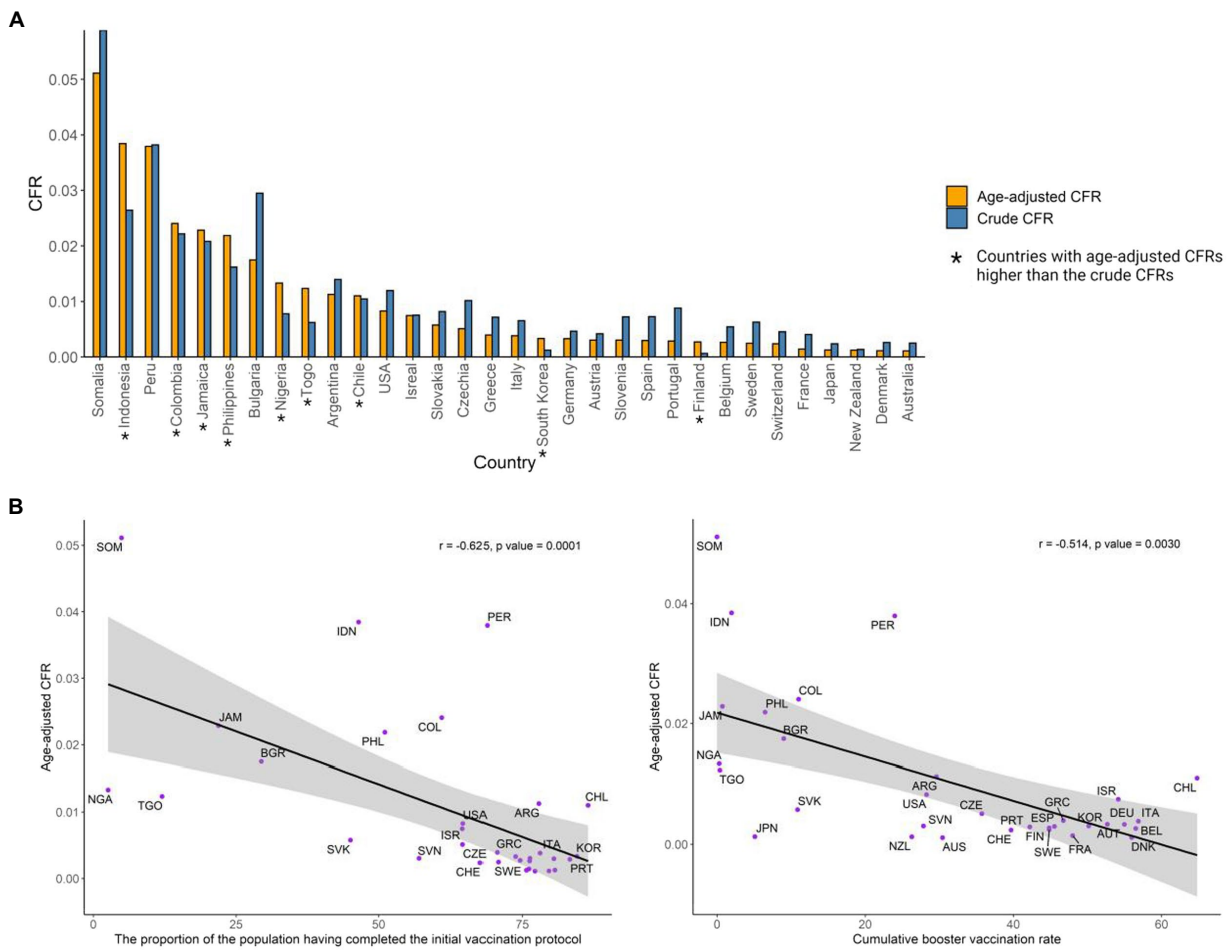
**(A)** Crude CFRs and age adjusted CFRs in the 32 countries. **(B)** The correlation between age-adjusted CFRs and vaccination coverage (fully vaccinated and booster given).

COVID-19 age-adjusted CFRs were available in 32 countries throughout the pandemic from February 4, 2020 to January 31, 2022. The crude CFRs in these countries ranged from 63 to 5,886 deaths per 100,000 people, and the age-adjusted CFRs still varied significantly across the country, ranging from 110 to 5,112 deaths per 100,000 people. The age-adjusted CFRs for the 32 countries during the original, Alpha, Delta, and Omicron periods were 1.25, 1.19, 1.37, and 0.16% respectively, showing a significant decrease for the Omicron period. According to the age-adjusted CFRs, the 32 countries were grouped into two groups: (1) Countries with higher age-adjusted CFRs than crude CFRs (*n* = 9, median age-adjusted and crude CFRs: 0.013 and 0.010), mainly in Asia and Africa; and (2) Countries with lower age-adjusted CFRs than crude CFRs (*n* = 23, median age-adjusted and crude CFRs: 0.003 and 0.007), mostly in Europe (Figure 2A). The countries with the highest age-adjusted CFR in group 1 are Indonesia, Colombia and Jamaica, and in group 2, they are Somalia, Peru and Bulgaria. The median cumulative full and booster vaccination rates for countries with higher age-adjusted CFRs than crude CFRs were 51.08 and 6.49%, respectively, while the median cumulative full and booster vaccination rates for countries with lower age-adjusted CFRs than crude CFRs were 73.83 and 39.7%, respectively. Univariate analyses revealed that age-adjusted CFRs were relatively strong associated with both the cumulative proportion of the population having completed the initial vaccination protocol and that having received a booster (*r* = −0.625, *p* = 0.0001; and *r* = −0.514, *p* = 0.0030, respectively) (Figure 2B). The higher the vaccination rate, the lower the CFRs, however, some countries with high vaccination rates, such as Peru, Chile and Colombia, still have relatively high CFRs.

### The determinants of age-adjusted CFRs over the pandemic

**Figure 3:**
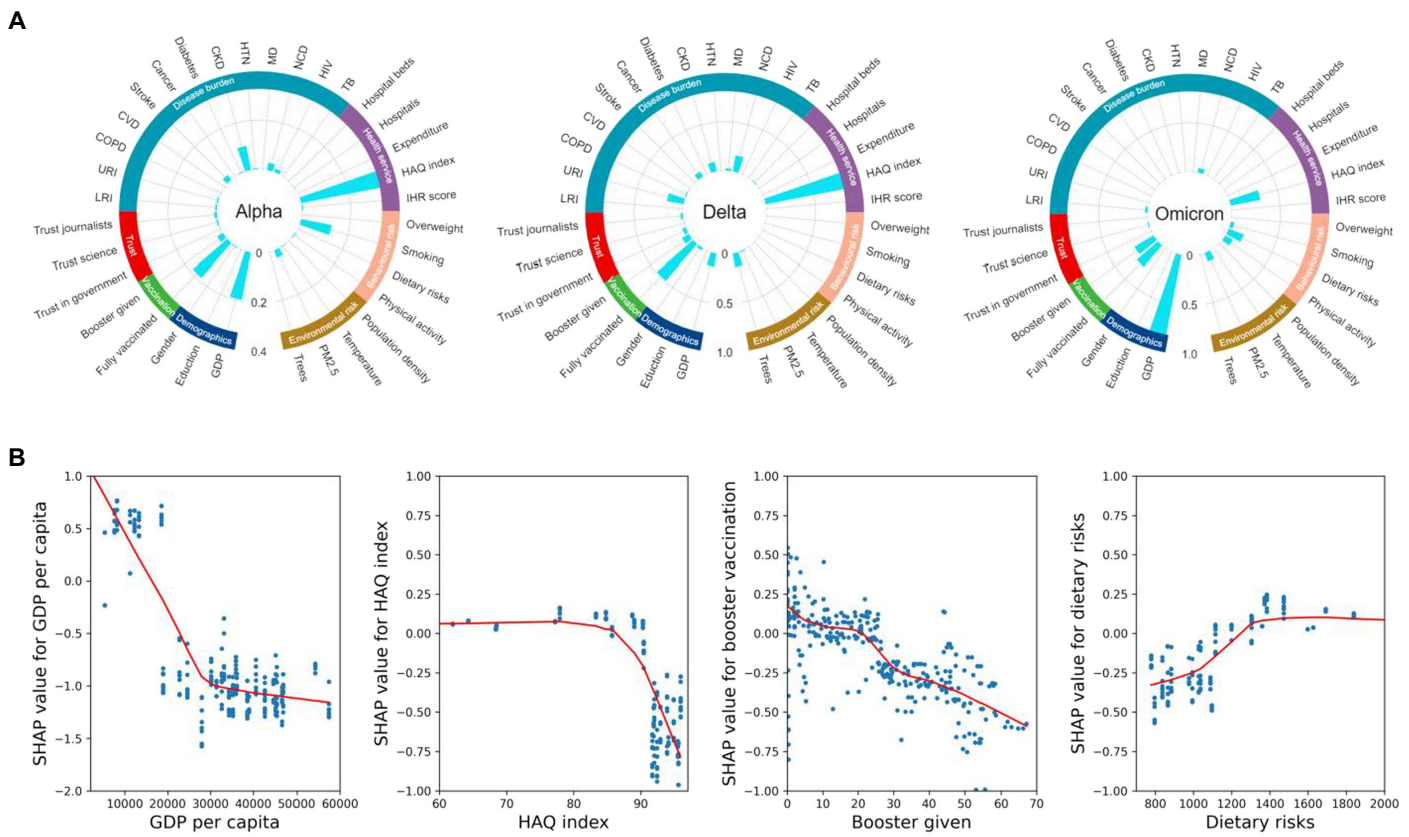
**(A)** Relative importance scores for each feature affecting age-adjusted CFR in each period model, obtained by taking the absolute mean of the SHAP values. The 35 features represent seven distinct dimensions: vaccination coverage, demographic factors, disease burden, behavioral risk factors, environmental risk factors, health services, and trust levels. **(B)** SHAP dependence plots for GDP *per capita*, HAQ index, booster vaccination rate, and dietary risks in the XGBoost models. SHAP values above zero represent an increased risk of higher COVID-19 age-adjusted CFR. LRI, lower respiratory infections; URI, upper respiratory infections; COPD, chronic obstructive pulmonary disease; CVD, cardiovascular diseases; CKD, chronic kidney disease; HTN, hypertension; MD, mental disorders; NCD, noncommunicable diseases; HIV, HIV infection; TB, tuberculosis.

Most cross-country variation in age-adjusted CFRs in the Alpha, Delta, and Omicron periods could be well explained by the SHAP-interpreted XGboost model (R^2^: 0.78, 0.88, 0.79, respectively) (Figure 3A). The XGboost-SHAP model showed that important determinants [importance score (IS) ≥ 0.10] in all three periods included HAQ index (IS: 0.36, 1.33 and 0.31 in the Alpha, Delta, and Omicron periods, respectively), GDP *per capita* (IS:0.19, 0.13, and 0.91) and vaccination (IS of fully vaccinated: 0.19, 0.49, and 0.28; IS of boost given: 0.03, 0.08, and 0.23), in addition to CKD (IS: 0.10), smoking (IS: 0.13) in the Alpha period, tree coverage (IS: 0.14), NCD (IS: 0.17), URI (IS: 0.17) in the Delta period, and dietary risks (IS: 0.17) in the Omicron period. Figure 3B showed that high booster vaccination rates and high GDP and HAQ indices are protective against age-adjusted CFR and that high dietary risks would be a risk for age-adjusted CFR in the Omicron period. Comparing the important determinants of CFR over the three periods shows that completing the initial vaccination protocol is more important in the Delta period (IS: 0.49), while the protective effects of booster vaccination increasingly become more important from Alpha to Omicron period (IS: 0.23; Figure 3B). Various underlying disease burdens were identified as important risk factors for CFR, such as chronic kidney disease (CKD) (IS: 0.10 in both Alpha and Delta period), and NCD (IS: 0.17 in the Delta period), but the risk posed by these underlying diseases was reduced for the Omicron period. In addition, high levels of trust in government, journalists and science are also protective factors for COVID-19 deaths in almost all periods. The importance of dietary risk on age-adjusted CFR is revealed in the Omicron period (IS: 0.17). PM2.5 as a risk factor in all periods became more important in the Omicron period (IS: 0.09).

### Assessment of country-specific risks for age adjusted CFR in the omicron period

**Figure 4:**
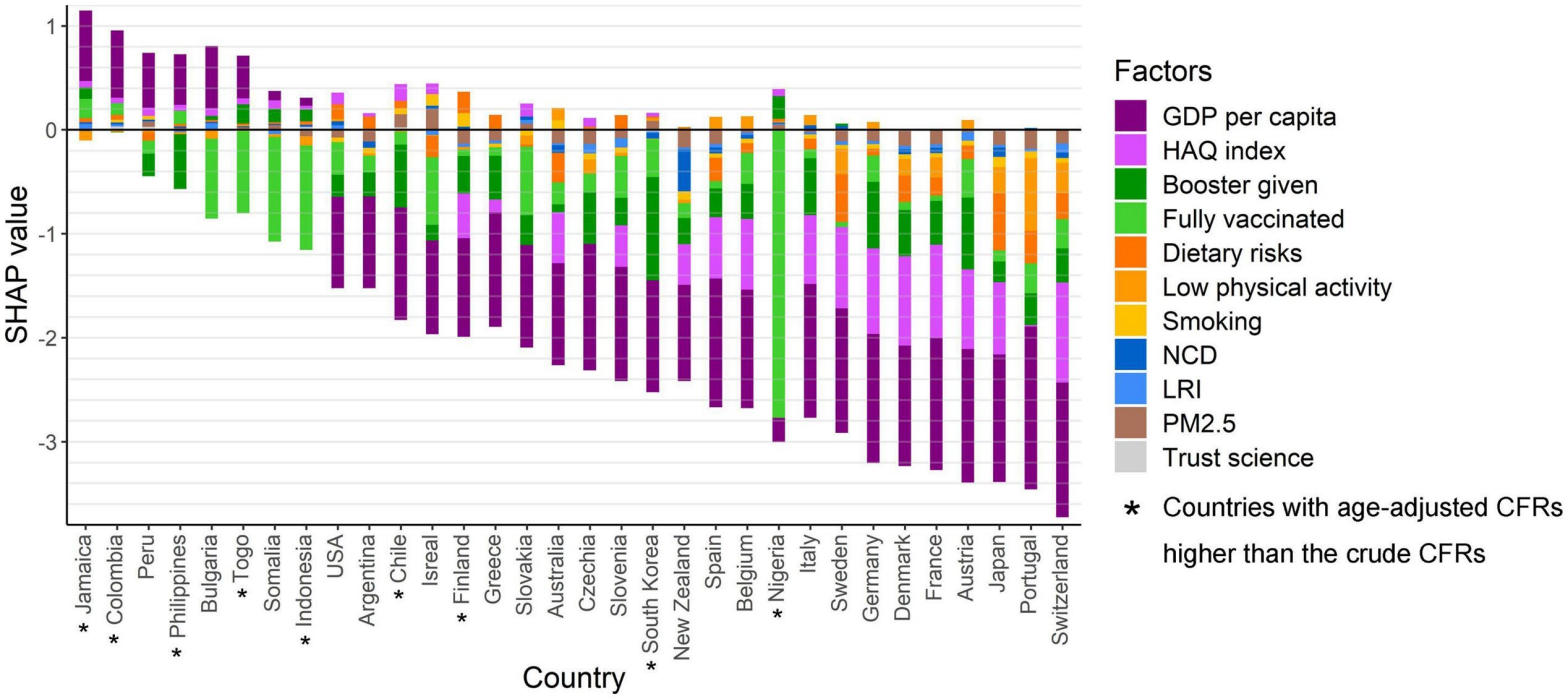
The protection and risk contributions of the determinants of the COVID-19 age-adjusted CFR for each country in the Omicron period. SHAP values above 0 are regarded as risk effects and below 0 as protective effects.

The determinants, including GDP *per capita*, HAQ index, vaccination coverage (population receiving booster doses and fully vaccinated) and behavioral risk factors (dietary risks, low physical activity, and smoking), disease burden (NCD and LRI), as well as PM2.5 and trust science, contribute to the COVID-19 age-adjusted CFR in each country during the Omicron period as shown in Figure 4. Countries are sorted from left to right in descending order of risk of death from COVID-19. The key risk factors for countries with higher age-adjusted CFR than crude CFR are low GDP *per capita* and low booster vaccination rates, while the key risk factors for countries with higher age-adjusted CFR than crude CFR were high dietary risks and low physical activity. Moreover, most countries were already protected by booster vaccination, but there were still seven countries (Sweden, Bulgaria, Jamaica, Indonesia, Somalia, Togo, Nigeria) with an increased risk of death from COVID-19 due to low booster vaccination rate. These countries have more concurrent risk factors, with all seven at risk of high NCD burden, six of the seven at risk of low HAQ index, and five of the seven at risk of low GDP *per capita*. Furthermore, high dietary risk and low physical activity also increased the risk of death from COVID-19 in 23 countries, with it being the main risk in 12 of these countries. The 11 of these 12 countries that are high-income countries already have a booster vaccination rate of 41.3%. CFRs are adversely affected by the burden of NCD to some degree in 65.6% of countries in this study, but of these only Portugal and Sweden have the main risk from NCD. In addition, high PM2.5 and low trust in science are the key risk factor in South Korea and Isreal, and Switzerland, respectively.

### Future benefits of increasing booster vaccination vary by country

Countries show varying degrees of reduction in age-adjusted CFRs when simulating 1–30% increase in booster vaccination (Supplementary Figure 2). Countries with age-adjusted CFRs higher than crude CFRs showed a reduction in CFRs when simulated booster vaccination rates were increased by 1–7%, and in addition, 11 of the countries with age-adjusted CFRs higher than crude CFRs (48%) also showed a reduction in CFRs. Furthermore, increasing booster vaccination for just up to 3% of the population would reduce CFR in 15 countries. These countries include five countries (Nigeria, Togo, Indonesia, Sweden, and Jamaica) with pre-existing low booster vaccination rates as a risk factor. However, Bulgaria, as a country where low vaccination rates are also a risk factor, would need to increase vaccination rates by 9% and above to bring down its CFR. Moreover, in Czechia, Australia and Portugal, the CFRs did not show a decrease until booster vaccination rates increased by more than 12%. Furthermore, in Austria, Belgium, Chile, Germany, Denmark, Italy, and Slovakia, the increase in booster vaccination did not significantly reduce the CFRs, where the average booster vaccination rate has reached 51.9%.

## Discussion

This is the first study to comprehensively identify risk factors affecting COVID-19 age-adjusted CFRs at the country level, particularly to assess and predict the effect of booster vaccination in the COVID-19 pandemic. Our models fit well allowing for a real-world assessment of the risk of COVID-19 death and the health benefits of vaccination in each country to more rationally guide vaccine distribution. We draw two conclusions from this study. First, booster vaccinations showed stronger importance in the Omicron period as previous vaccine effectiveness waned, while the importance of other factors such as disease burden and behavioral risk factors for CFR changed during the pandemic. Our study confirms the importance of vaccination, especially booster doses, in reducing the risk of death in Omicron pandemics. Patients during the Omicron period also benefited from the strong protection against severe disease and death still afforded by the COVID-19 vaccine (50). In the stage dominated by the “Stealth” Omicron, during which strict prevention policies are challenged by insidious transmission and the number of infections has become difficult to control, improving vaccination coverage is a cost-effective approach for reducing severe health outcomes and relieving pressure on the healthcare system. On the issue of vaccine allocation, as advocated by Jeremy Bentham’s Utilitarianism, a rule for society should be established that has the best outcome for the greatest amount of people in society, in the sense that a cost-effective vaccine allocation scheme should be developed in a global perspective that reduces the risk of death for the greatest proportion of people worldwide. Our study simulated the reduction in CFR after increasing vaccination by country and found that the health benefits of increasing vaccination varied by country, for example, countries such as Togo, Isreal, and Nigeria showed significant reductions in CFR with only a small increase in vaccination. The WHO has worked to this end by convening COVAX (51), a ground-breaking global collaboration aimed at accelerating the development and production of and equitable access to the COVID-19 vaccine, ensuring that every country has access to the vaccine and is able to promote vaccination to protect their whole population, starting with the most vulnerable. On the other hand, GDP *per capita* and HAQ index have been important determinants of age-adjusted CFR during the different variant-dominated periods of the pandemic. The HAQ index reflects the accessibility and quality of health care for individuals. The accessibility and quality of healthcare in a country are important when responding to a pandemic; moreover, regional inequities in access and quality may lead to greater regional disparities in the burden of infectious diseases in the future. Adjusting investments to improve access and quality across healthcare needs will not only benefit routine care, but also improve overall health coverage in preparation for the next pandemic (43). For instance, our study presented that there were several countries (e.g., Sweden, Bulgaria, Jamaica, Indonesia, Somalia, Togo, Nigeria) have low GDP and HAQ indices, as well as low boost vaccination coverage, which contribute to their high risk of death.

The second major conclusion of this study is that CFRs are also affected by a broad range of concurrent risks, such as high dietary risk, low physical activity, high disease burden, and high PM2.5. Consequently, we believe that a joint intervention would be an effective measure for reducing CFRs in this class of countries. In the short term, in addition to vaccination, a promising area for interventionists to work on is raising the level of national trust. Our findings support previous research that trust in government and science can increase risk perceptions of COVID-19 among the population, promote cooperation with outbreak prevention and control efforts, and more quickly control the number of cases and deaths (52). Pandemics have always posed a challenge to trust between the public and the government, and maintaining and rebuilding trust during a crisis is crucial to maintaining political participation and social cohesion (53). In the long term, behavioral factors such as smoking, diet, and nutrition, along with environmental factors such as PM2.5, are all risk factors that can be changed through health education and policy development, and are areas in which advanced preparation is needed in order to mitigate the effects of future epidemics. In high-income countries, dietary risks are revealed. Dietary risk is the intake of too much or too little of certain foods or nutrients. As studies have shown, a healthy dietary pattern is associated with lower risk and severity of COVID-19 (54). Therefore, improving the dietary health of the population or correcting micronutrient deficiencies in people already diagnosed with COVID-19 infection may help to reduce the risk of death (55). Moreover, regulating taxes on tobacco, tightening restrictions on smoking places, and setting a legal age for smoking would contribute to reducing the potential harm from smoking at a national level. In addition, environmental factors are of increasing concern to epidemiologists, and our research suggests that PM2.5 have some impact on severe health outcomes in COVID-19. It has also been suggested that PM2.5 may potentially serve as a carrier for the virus (56). Therefore, an improved environment with less air pollution would benefit both patients with COVID-19 and healthy populations. Consequently, we believe that a joint intervention would be an effective measure for reducing CFRs in the countries.

There are several limitations in our analysis. First, the study design is a country-level ecological analysis based on retrospective data, and care should be taken regarding ecological fallacies in the interpretation and generalization of the results. Second, our data were sourced from multiple publicly available data sources, and after comparing them we selected the more credible sources and also applied outlier treatment, but the credibility of our analysis relies greatly on the quality of the data. Third, COVID-19 cases and deaths are from national self-reported data and do not consider excess deaths from COVID-19. Fourth, we predicted the future CFR only when increasing booster vaccination rates in each country, keeping other factors constant, considering that only vaccination rates are relatively changeable in the short term among the factors that affect CFR. Fifth, some of the potential factors affecting CFR were not available in this study, such as vaccine type and ethnicity, and in addition there may be incorrect estimates based on missing values due to the missing values in the data.

In conclusion, the cross-temporal and cross-country variation in COVID-19 age-adjusted CFRs illustrates the importance of conducting further research on risk assessment. The future health benefits of increased vaccination are country-specific due to differences in risk factors of CFR by country. Booster vaccination still plays an important role in reducing age-adjusted CFRs, while there are multidimensional concurrent risk factors and precise joint intervention strategies and preparations based on country-specific risks are also essential. Our study reminds policy makers to consider risk factors holistically and assess whether their countries can rebuild policy trust, face the challenges of vaccine hesitancy, revitalize primary healthcare, and strengthen behavioral and environmental risk management and investment in the post-COVID era.

## Data availability statement

The original contributions presented in the study are included in the article/Supplementary material, further inquiries can be directed to the corresponding authors.

## Author contributions

CuZ: data collection, conceptualization, investigation, data analysis, and writing original draft and revision. ÅW: conceptualization, supervision, and investigation. ChZ, JM, and ZL: data analysis. KD and JP: revision. WL: conceptualization and supervision. JG: data collection, conceptualization, supervision, investigation, data analysis, and writing original draft and revision. LX: conceptualization, supervision, and funding acquisition, writing, and revision. All authors contributed to the article and approved the submitted version.

## Funding

This study received support from the National Key R&D Program of China (Grant No. 2021ZD0114103) and Karolinska Institutet Research Foundation Grants (2022-02329). We also extend our thanks to the Research Fund at Vanke School of Public Health, Tsinghua University, China, for their support. The funder has no role in the data collection, data analysis, preparation of manuscript and decision to submission.

## Conflict of interest

The authors declare that the research was conducted in the absence of any commercial or financial relationships that could be construed as a potential conflict of interest.

## Publisher’s note

All claims expressed in this article are solely those of the authors and do not necessarily represent those of their affiliated organizations, or those of the publisher, the editors and the reviewers. Any product that may be evaluated in this article, or claim that may be made by its manufacturer, is not guaranteed or endorsed by the publisher.
