# Supplementary material for: The role of booster vaccination in decreasing COVID-19 age-adjusted case fatality rate: Evidence from 32 countries
Source: Front Public Health. 2023 Apr 18;11:1150095. doi: 10.3389/fpubh.2023.1150095 (PMC10151823; doi:10.3389/fpubh.2023.1150095)

Supplementary Material

**Abbreviations**

COVID-19: coronavirus disease 2019

SARS-CoV-2: severe acute respiratory syndrome coronavirus 2

CFRs: case fatality rates

VOC: variant of concern

XGBoost: Extreme Gradient Boosting

LASSO: least absolute shrinkage and selection operator

SHAP: SHapley Additive exPlanations

WHO: World Health Organization

RFE: recursive feature elimination

RMSE: root-mean-square error

SD: standard deviation

IQR: interquartile range

ANOVA: analysis of variance

HAQ Index: Healthcare Access and Quality Index

IHR: International Health Regulations core capacity

GDP: gross domestic product

LRI: lower respiratory infections

URI: upper respiratory infections

COPD: chronic obstructive pulmonary disease

CVD: cardiovascular diseases

CKD: chronic kidney disease

HTN: hypertension

MD: mental disorders

NCD: noncommunicable diseases

HIV: HIV infection

TB: tuberculosis

**Supplement****ary table 1. The list of** **covariates used in analyses.**

|  | **Dimension** | **Definition** | **Temporal coverage** | **Data source** | **Missing** | **Median (IQR)** |
| --- | --- | --- | --- | --- | --- | --- |
| Fully vaccinated | Vaccination coverage | Proportion of the population completing the initial vaccination protocol within six months | 2020-2022 | Our World in Data | 0/156 | 27.3 (11.4-40.0) |
| Booster given |  | Proportion of the population that received a booster dose within six months | 2020-2022 | Our World in Data | 0/156 | 3.9 (0-18.6) |
| Gender ratio |  | Number of males born per 100 females | 2020 | World Development Indicators - World Bank | 0/156 | 105.1 (103.9-105.9) |
| Average years of schooling |  | Average number of years the population older than 25 participated in formal education | 2017 | Our World in Data | 2/156 | 9.2 (6.3-11.3) |
| GDP per capita |  | Per capita gross domestic product, a measure of a country's economic output per person | 2018 | World Bank | 4/156 | 13567.3 (4796.5-28942.6) |
| Lower respiratory infections | Disease burden | Age-standardised prevalence of lower respiratory infections per 100,000 population | 2019 | Global Burden of Disease Study 2019 | 0/156 | 130.7 (84.2-184.6) |
| Upper respiratory infections |  | Age-standardised prevalence of upper respiratory infections per 100,000 population | 2019 | Global Burden of Disease Study 2019 | 0/156 | 3228 (2774-3712) |
| Chronic obstructive pulmonary disease |  | Age-standardised prevalence of chronic obstructive pulmonary disease per 100,000 population | 2019 | Global Burden of Disease Study 2019 | 0/156 | 2054.3 (1713.3-2665.0) |
| Cardiovascular diseases |  | Age-standardised prevalence of cardiovascular diseases per 100,000 population | 2019 | Global Burden of Disease Study 2019 | 0/156 | 6477 (5616-7132) |
| Stroke |  | Age-standardised prevalence of stroke per 100,000 population | 2019 | Global Burden of Disease Study 2019 | 0/156 | 1233.6 (958.2-1411.3) |
| Cancers |  | Age-standardised prevalence of cancers per 100,000 population | 2019 | Global Burden of Disease Study 2019 | 0/156 | 6157 (4421-9517) |
| Diabetes |  | Age-standardised prevalence of diabetes per 100,000 population | 2019 | Global Burden of Disease Study 2019 | 0/156 | 5490 (4243-7292) |
| Chronic kidney disease |  | Age-standardised prevalence of chronic kidney disease per 100,000 population | 2019 | Global Burden of Disease Study 2019 | 0/156 | 7871 (6384-9957) |
| Hypertension |  | Prevalence of hypertension among adults aged 30-79 years | 2019 | Global Burden of Disease Study 2019 | 1/156 | 37.3 (31.6-42.1) |
| Mental disorders |  | Age-standardised prevalence of mental disorders per 100,000 population | 2019 | Global Burden of Disease Study 2019 | 0/156 | 12651 (11365-13971) |
| Noncommunicable diseases |  | Age-standardised total mortality from noncommunicable diseases per 100,000 population | 2019 | WHO | 0/156 | 90447 (89596-91749) |
| HIV |  | Age-standardised prevalence of HIV infection per 100,000 population | 2019 | Global Burden of Disease Study 2019 | 0/156 | 176.8 (46.4-589.7) |
| Tuberculosis |  | Age-standardised prevalence of tuberculosis per 100,000 population | 2019 | Global Burden of Disease Study 2019 | 0/156 | 19036 (13148-24781) |
| Overweight | Behavioural risk factor | Prevalence of overweight among adults, BMI ≥ 25 (age-standardized estimate) (%) | 2016 | WHO | 2/156 | 55.6 (30.9-60.6) |
| Low physical activity |  | Disability-adjusted life years attributed to low physical activity | 2019 | Global Burden of Disease Study 2019 | 0/156 | 188.2 (134.2-330.3) |
| Smoking |  | Disability-adjusted life years attributed to smoking | 2019 | Global Burden of Disease Study 2019 | 0/156 | 2024.9 (1517.8-2721.2) |
| Dietary risks |  | Disability-adjusted life years attributed to dietary risks | 2019 | Global Burden of Disease Study 2019 | 0/156 | 2210.2 (1607.3-2950.3) |
| Trees per capita | Environmental risk factor | Number of trees per capita | 2014 | Mapping tree density at a global scale | 0/156 | 191.0 (52.2-695.8) |
| PM2.5 |  | Population-weighted exposure to ambient PM2.5 pollution (μg/m3) is defined as the average level of exposure of a nation's population to concentrations of suspended particles measuring less than 2.5 microns in aerodynamic diameter, which are capable of penetrating deep into the respiratory tract and causing severe health damage | 2017 | Global Burden of Disease Study 2019 | 1/156 | 22.2 (14.5-36.7) |
| Mean temperature |  | Mean temperature (Celsius) | 1991-2020 | World Bank Climate Change Knowledge Portal | 0/156 | 22.1 (11.3-25.5) |
| Population density |  | Population density (people per sq. km of land area) | 2020 | World Bank | 1/156 | 81.7 (31.8-160.8) |
| HAQ index | Health service | Healthcare Access and Quality Index | 2018 | GBD 2016 Healthcare Access and Quality Collaborators | 8/156 | 66.8 (41.5-81.0) |
| IHR score |  | Average of 13 International Health Regulations core capacity scores, 1st version of the questionnaire | 2019 | WHO | 0/156 | 66.5 (49.8-83.0) |
| Hospital beds |  | The number of hospital beds available per 10,000 inhabitants | 2017 | WHO | 3/156 | 20.6 (10.0-36.0) |
| Expenditure |  | Current health expenditure per capita in US$ | 2019 | WHO | 4/156 | 392.5 (75.2-1407.3) |
| Hospitals |  | Total density of hospitals per 100,000 population | 2013 | WHO | 49/156 | 1.0 (0.5-1.8) |
| Trust the national government | Trust | Trust in the national government of the country | 2020 | Wellcome Global Monitor Survey | 55/156 | 52.5 (43.0-69.5) |
| Trust journalists |  | Trust in journalists in this country | 2020 | Wellcome Global Monitor Survey | 51/156 | 55.2 (47.4-64.8) |
| Trust science |  | Trust in science | 2020 | Wellcome Global Monitor Survey | 50/156 | 80.5 (70.0-88.8) |

Abbreviations: GDP, gross domestic product; PM2.5: particles measuring less than 2.5 microns in aerodynamic diameter; HAQ, Healthcare Access and Quality Index; IHR, International Health Regulations core capacity; WHO, World Health Organization.

**Supplementary table 2. Results of feature selection using RFE**

1. Feature selection of Alpha period model

| **Number of variable** | **RMSE (per 1,000,000 population)** | **Variables** |
| --- | --- | --- |
| 31 | 8756.214372 | ['HAQ.index', 'NCD', 'GDP.per.capita', 'Fully.vaccinated', 'Smoking', 'HIV', 'Stroke', 'PM2.5', 'Trees.per.capita', 'Trust.science', 'Dietary.risks', 'Booster', 'Trust.journalists', 'Chronic.kidney.disease', 'IHR.score', 'Expenditure', 'Overweight', 'Hospitals', 'Tuberculosis', 'Diabetes', 'Gender.ratio', 'Low.physical.activity', 'Upper.respiratory.infections', 'COPD', 'Cardiovascular.diseases', 'Cancers', 'Hypertension', 'Average.years.of.schooling', 'Trust.the.national.government', 'Temperature', 'Lower.respiratory.infections'] |
| 21 | 8764.465246 | ['HAQ.index', 'NCD', 'GDP.per.capita', 'Fully.vaccinated', 'Smoking', 'HIV', 'Stroke', 'PM2.5', 'Trees.per.capita', 'Trust.science', 'Dietary.risks', 'Booster', 'Trust.journalists', 'Chronic.kidney.disease', 'IHR.score', 'Expenditure', 'Overweight', 'Hospitals', 'Tuberculosis', 'Diabetes', 'Gender.ratio'] |
| 25 | 8782.505239 | ['HAQ.index', 'NCD', 'GDP.per.capita', 'Fully.vaccinated', 'Smoking', 'HIV', 'Stroke', 'PM2.5', 'Trees.per.capita', 'Trust.science', 'Dietary.risks', 'Booster', 'Trust.journalists', 'Chronic.kidney.disease', 'IHR.score', 'Expenditure', 'Overweight', 'Hospitals', 'Tuberculosis', 'Diabetes', 'Gender.ratio', 'Low.physical.activity', 'Upper.respiratory.infections', 'COPD', 'Cardiovascular.diseases'] |
| 26 | 8787.879206 | ['HAQ.index', 'NCD', 'GDP.per.capita', 'Fully.vaccinated', 'Smoking', 'HIV', 'Stroke', 'PM2.5', 'Trees.per.capita', 'Trust.science', 'Dietary.risks', 'Booster', 'Trust.journalists', 'Chronic.kidney.disease', 'IHR.score', 'Expenditure', 'Overweight', 'Hospitals', 'Tuberculosis', 'Diabetes', 'Gender.ratio', 'Low.physical.activity', 'Upper.respiratory.infections', 'COPD', 'Cardiovascular.diseases', 'Cancers'] |
| 20 | 8789.047545 | ['HAQ.index', 'NCD', 'GDP.per.capita', 'Fully.vaccinated', 'Smoking', 'HIV', 'Stroke', 'PM2.5', 'Trees.per.capita', 'Trust.science', 'Dietary.risks', 'Booster', 'Trust.journalists', 'Chronic.kidney.disease', 'IHR.score', 'Expenditure', 'Overweight', 'Hospitals', 'Tuberculosis', 'Diabetes'] |
| 19 | 8798.269248 | ['HAQ.index', 'NCD', 'GDP.per.capita', 'Fully.vaccinated', 'Smoking', 'HIV', 'Stroke', 'PM2.5', 'Trees.per.capita', 'Trust.science', 'Booster', 'Dietary.risks', 'Trust.journalists', 'Chronic.kidney.disease', 'IHR.score', 'Expenditure', 'Overweight', 'Hospitals', 'Tuberculosis'] |
| 17 | 8802.188955 | ['HAQ.index', 'NCD', 'GDP.per.capita', 'Fully.vaccinated', 'Smoking', 'HIV', 'PM2.5', 'Trees.per.capita', 'Stroke', 'Booster', 'Dietary.risks', 'Trust.science', 'Trust.journalists', 'Chronic.kidney.disease', 'IHR.score', 'Hospitals', 'Overweight'] |
| 32 | 8864.24919 | ['HAQ.index', 'NCD', 'GDP.per.capita', 'Fully.vaccinated', 'Smoking', 'HIV', 'Stroke', 'PM2.5', 'Trees.per.capita', 'Trust.science', 'Dietary.risks', 'Booster', 'Trust.journalists', 'Chronic.kidney.disease', 'Expenditure', 'IHR.score', 'Hospitals', 'Overweight', 'Tuberculosis', 'Diabetes', 'Gender.ratio', 'Low.physical.activity', 'Upper.respiratory.infections', 'COPD', 'Cardiovascular.diseases', 'Cancers', 'Hypertension', 'Average.years.of.schooling', 'Trust.the.national.government', 'Temperature', 'Lower.respiratory.infections', 'Hospital.beds'] |
| 18 | 8864.776558 | ['HAQ.index', 'NCD', 'GDP.per.capita', 'Fully.vaccinated', 'Smoking', 'HIV', 'PM2.5', 'Trees.per.capita', 'Stroke', 'Trust.science', 'Dietary.risks', 'Booster', 'Trust.journalists', 'Chronic.kidney.disease', 'Overweight', 'IHR.score', 'Hospitals', 'Expenditure'] |
| 12 | 8871.806763 | ['HAQ.index', 'NCD', 'GDP.per.capita', 'Fully.vaccinated', 'Smoking', 'HIV', 'PM2.5', 'Trees.per.capita', 'Dietary.risks', 'Stroke', 'Trust.science', 'Booster'] |
| 6 | 8875.603873 | ['HAQ.index', 'NCD', 'GDP.per.capita', 'Fully.vaccinated', 'Smoking', 'HIV'] |
| 22 | 8878.276434 | ['HAQ.index', 'NCD', 'GDP.per.capita', 'Fully.vaccinated', 'Smoking', 'HIV', 'Stroke', 'PM2.5', 'Trees.per.capita', 'Trust.science', 'Dietary.risks', 'Booster', 'Trust.journalists', 'Chronic.kidney.disease', 'IHR.score', 'Expenditure', 'Overweight', 'Hospitals', 'Tuberculosis', 'Diabetes', 'Gender.ratio', 'Low.physical.activity'] |
| 11 | 8878.368348 | ['HAQ.index', 'NCD', 'GDP.per.capita', 'Fully.vaccinated', 'Smoking', 'HIV', 'PM2.5', 'Stroke', 'Trust.science', 'Trees.per.capita', 'Dietary.risks'] |
| 5 | 8888.109157 | ['HAQ.index', 'NCD', 'GDP.per.capita', 'Fully.vaccinated', 'Smoking'] |
| 24 | 8891.099191 | ['HAQ.index', 'NCD', 'GDP.per.capita', 'Fully.vaccinated', 'Smoking', 'HIV', 'Stroke', 'PM2.5', 'Trees.per.capita', 'Trust.science', 'Dietary.risks', 'Booster', 'Trust.journalists', 'Chronic.kidney.disease', 'IHR.score', 'Expenditure', 'Overweight', 'Hospitals', 'Tuberculosis', 'Diabetes', 'Gender.ratio', 'Low.physical.activity', 'Upper.respiratory.infections', 'COPD'] |
| 14 | 8891.588162 | ['HAQ.index', 'NCD', 'GDP.per.capita', 'Fully.vaccinated', 'Smoking', 'HIV', 'Trust.science', 'Trees.per.capita', 'Stroke', 'PM2.5', 'Booster', 'Trust.journalists', 'Dietary.risks', 'Chronic.kidney.disease'] |
| 23 | 8892.819075 | ['HAQ.index', 'NCD', 'GDP.per.capita', 'Fully.vaccinated', 'Smoking', 'HIV', 'Stroke', 'PM2.5', 'Trees.per.capita', 'Trust.science', 'Dietary.risks', 'Booster', 'Trust.journalists', 'Chronic.kidney.disease', 'IHR.score', 'Expenditure', 'Overweight', 'Hospitals', 'Tuberculosis', 'Diabetes', 'Gender.ratio', 'Low.physical.activity', 'Upper.respiratory.infections'] |
| 10 | 8895.175384 | ['HAQ.index', 'NCD', 'GDP.per.capita', 'Fully.vaccinated', 'Smoking', 'HIV', 'PM2.5', 'Trees.per.capita', 'Dietary.risks', 'Stroke'] |
| 16 | 8906.756222 | ['HAQ.index', 'NCD', 'GDP.per.capita', 'Fully.vaccinated', 'Smoking', 'HIV', 'PM2.5', 'Trees.per.capita', 'Stroke', 'Trust.science', 'Booster', 'Dietary.risks', 'Trust.journalists', 'Chronic.kidney.disease', 'IHR.score', 'Overweight'] |
| 9 | 8907.544298 | ['HAQ.index', 'NCD', 'GDP.per.capita', 'Fully.vaccinated', 'Smoking', 'HIV', 'PM2.5', 'Trees.per.capita', 'Dietary.risks'] |
| 30 | 8912.199961 | ['HAQ.index', 'NCD', 'GDP.per.capita', 'Fully.vaccinated', 'Smoking', 'HIV', 'Stroke', 'PM2.5', 'Trees.per.capita', 'Trust.science', 'Dietary.risks', 'Booster', 'Trust.journalists', 'Chronic.kidney.disease', 'IHR.score', 'Expenditure', 'Overweight', 'Hospitals', 'Tuberculosis', 'Diabetes', 'Gender.ratio', 'Low.physical.activity', 'Upper.respiratory.infections', 'COPD', 'Cardiovascular.diseases', 'Cancers', 'Hypertension', 'Average.years.of.schooling', 'Trust.the.national.government', 'Temperature'] |
| 13 | 8912.836215 | ['HAQ.index', 'NCD', 'GDP.per.capita', 'Fully.vaccinated', 'Smoking', 'HIV', 'PM2.5', 'Trees.per.capita', 'Stroke', 'Dietary.risks', 'Trust.science', 'Trust.journalists', 'Booster'] |
| 29 | 8935.591787 | ['HAQ.index', 'NCD', 'GDP.per.capita', 'Fully.vaccinated', 'Smoking', 'HIV', 'Stroke', 'PM2.5', 'Trees.per.capita', 'Trust.science', 'Dietary.risks', 'Booster', 'Trust.journalists', 'Chronic.kidney.disease', 'IHR.score', 'Expenditure', 'Overweight', 'Hospitals', 'Tuberculosis', 'Diabetes', 'Gender.ratio', 'Low.physical.activity', 'Upper.respiratory.infections', 'COPD', 'Cardiovascular.diseases', 'Cancers', 'Hypertension', 'Average.years.of.schooling', 'Trust.the.national.government'] |
| 27 | 8943.306867 | ['HAQ.index', 'NCD', 'GDP.per.capita', 'Fully.vaccinated', 'Smoking', 'HIV', 'Stroke', 'PM2.5', 'Trees.per.capita', 'Trust.science', 'Dietary.risks', 'Booster', 'Trust.journalists', 'Chronic.kidney.disease', 'IHR.score', 'Expenditure', 'Overweight', 'Hospitals', 'Tuberculosis', 'Diabetes', 'Gender.ratio', 'Low.physical.activity', 'Upper.respiratory.infections', 'COPD', 'Cardiovascular.diseases', 'Cancers', 'Hypertension'] |
| 8 | 8961.393878 | ['HAQ.index', 'NCD', 'GDP.per.capita', 'Fully.vaccinated', 'Smoking', 'HIV', 'Dietary.risks', 'PM2.5'] |
| 7 | 8963.142599 | ['HAQ.index', 'NCD', 'GDP.per.capita', 'Fully.vaccinated', 'Smoking', 'HIV', 'Dietary.risks'] |
| 15 | 8963.677742 | ['HAQ.index', 'NCD', 'GDP.per.capita', 'Fully.vaccinated', 'HIV', 'Smoking', 'Trust.science', 'Trees.per.capita', 'Stroke', 'PM2.5', 'Dietary.risks', 'Trust.journalists', 'Booster', 'Chronic.kidney.disease', 'IHR.score'] |
| 28 | 8970.737237 | ['HAQ.index', 'NCD', 'GDP.per.capita', 'Fully.vaccinated', 'Smoking', 'HIV', 'Stroke', 'PM2.5', 'Trees.per.capita', 'Trust.science', 'Dietary.risks', 'Booster', 'Trust.journalists', 'Chronic.kidney.disease', 'IHR.score', 'Expenditure', 'Overweight', 'Hospitals', 'Tuberculosis', 'Diabetes', 'Gender.ratio', 'Low.physical.activity', 'Upper.respiratory.infections', 'COPD', 'Cardiovascular.diseases', 'Cancers', 'Hypertension', 'Average.years.of.schooling'] |
| 33 | 9049.135987 | ['HAQ.index', 'NCD', 'GDP.per.capita', 'Fully.vaccinated', 'Smoking', 'HIV', 'Stroke', 'Overweight', 'PM2.5', 'Trust.science', 'Temperature', 'Trees.per.capita', 'Gender.ratio', 'Booster', 'Dietary.risks', 'Trust.journalists', 'IHR.score', 'Expenditure', 'Chronic.kidney.disease', 'Hospitals', 'Tuberculosis', 'Diabetes', 'Hospital.beds', 'Lower.respiratory.infections', 'Hypertension', 'Trust.the.national.government', 'Average.years.of.schooling', 'Low.physical.activity', 'Cancers', 'Cardiovascular.diseases', 'COPD', 'Upper.respiratory.infections', 'Population.density'] |

1. Feature selection of Delta period model

| **Number of variable** | **RMSE (per 1,000,000 population)** | **Variables** |
| --- | --- | --- |
| 20 | 9786.747498 | ['HAQ.index', 'Fully.vaccinated', 'NCD', 'GDP.per.capita', 'Trust.the.national.government', 'Booster', 'Chronic.kidney.disease', 'Cancers', 'Gender.ratio', 'Trust.science', 'PM2.5', 'Hospital.beds', 'Upper.respiratory.infections', 'Overweight', 'Mental.disorders', 'COPD', 'IHR.score', 'Low.physical.activity', 'Trust.journalists', 'Trees.per.capita'] |
| 26 | 9836.106118 | ['HAQ.index', 'Fully.vaccinated', 'NCD', 'GDP.per.capita', 'Trust.the.national.government', 'Booster', 'Chronic.kidney.disease', 'Cancers', 'Upper.respiratory.infections', 'Gender.ratio', 'Hospital.beds', 'PM2.5', 'Mental.disorders', 'IHR.score', 'Trust.science', 'Overweight', 'COPD', 'Low.physical.activity', 'Hypertension', 'Diabetes', 'Dietary.risks', 'Population.density', 'Trust.journalists', 'Trees.per.capita', 'Expenditure', 'Tuberculosis'] |
| 21 | 9867.83083 | ['HAQ.index', 'Fully.vaccinated', 'NCD', 'GDP.per.capita', 'Trust.the.national.government', 'Booster', 'Chronic.kidney.disease', 'Cancers', 'Gender.ratio', 'Upper.respiratory.infections', 'Hospital.beds', 'Trust.science', 'Mental.disorders', 'Overweight', 'COPD', 'IHR.score', 'PM2.5', 'Low.physical.activity', 'Trust.journalists', 'Trees.per.capita', 'Hypertension'] |
| 22 | 9883.849356 | ['HAQ.index', 'Fully.vaccinated', 'NCD', 'GDP.per.capita', 'Trust.the.national.government', 'Booster', 'Chronic.kidney.disease', 'Cancers', 'Gender.ratio', 'Trust.science', 'Upper.respiratory.infections', 'Hospital.beds', 'Overweight', 'Mental.disorders', 'COPD', 'PM2.5', 'IHR.score', 'Low.physical.activity', 'Trust.journalists', 'Trees.per.capita', 'Hypertension', 'Dietary.risks'] |
| 25 | 9919.81721 | ['HAQ.index', 'Fully.vaccinated', 'NCD', 'GDP.per.capita', 'Trust.the.national.government', 'Booster', 'Chronic.kidney.disease', 'Cancers', 'Upper.respiratory.infections', 'Gender.ratio', 'Hospital.beds', 'PM2.5', 'Mental.disorders', 'IHR.score', 'Trust.science', 'Overweight', 'COPD', 'Low.physical.activity', 'Hypertension', 'Diabetes', 'Dietary.risks', 'Population.density', 'Trust.journalists', 'Trees.per.capita', 'Expenditure'] |
| 19 | 9941.361285 | ['HAQ.index', 'Fully.vaccinated', 'NCD', 'GDP.per.capita', 'Trust.the.national.government', 'Booster', 'Chronic.kidney.disease', 'Cancers', 'Upper.respiratory.infections', 'Overweight', 'PM2.5', 'Hospital.beds', 'Gender.ratio', 'Trust.science', 'COPD', 'Mental.disorders', 'IHR.score', 'Trees.per.capita', 'Trust.journalists'] |
| 23 | 9947.915006 | ['HAQ.index', 'Fully.vaccinated', 'NCD', 'GDP.per.capita', 'Trust.the.national.government', 'Booster', 'Chronic.kidney.disease', 'Cancers', 'Gender.ratio', 'Hospital.beds', 'Overweight', 'Upper.respiratory.infections', 'IHR.score', 'PM2.5', 'COPD', 'Trust.science', 'Mental.disorders', 'Low.physical.activity', 'Hypertension', 'Trees.per.capita', 'Trust.journalists', 'Dietary.risks', 'Expenditure'] |
| 24 | 9971.032609 | ['HAQ.index', 'Fully.vaccinated', 'NCD', 'GDP.per.capita', 'Trust.the.national.government', 'Booster', 'Chronic.kidney.disease', 'Cancers', 'Gender.ratio', 'Hospital.beds', 'Overweight', 'Upper.respiratory.infections', 'IHR.score', 'PM2.5', 'COPD', 'Trust.science', 'Mental.disorders', 'Low.physical.activity', 'Hypertension', 'Trust.journalists', 'Trees.per.capita', 'Dietary.risks', 'Expenditure', 'Diabetes'] |
| 17 | 9982.615102 | ['HAQ.index', 'Fully.vaccinated', 'NCD', 'GDP.per.capita', 'Trust.the.national.government', 'Chronic.kidney.disease', 'Booster', 'Upper.respiratory.infections', 'Cancers', 'Hospital.beds', 'Gender.ratio', 'IHR.score', 'PM2.5', 'Mental.disorders', 'Trust.journalists', 'Trust.science', 'Overweight'] |
| 32 | 10021.90021 | ['HAQ.index', 'Fully.vaccinated', 'NCD', 'GDP.per.capita', 'Trust.the.national.government', 'Booster', 'Chronic.kidney.disease', 'Cancers', 'Upper.respiratory.infections', 'Gender.ratio', 'Hospital.beds', 'PM2.5', 'Mental.disorders', 'IHR.score', 'Trust.science', 'Overweight', 'COPD', 'Diabetes', 'Low.physical.activity', 'Hypertension', 'Dietary.risks', 'Population.density', 'Trust.journalists', 'Trees.per.capita', 'Expenditure', 'Tuberculosis', 'Lower.respiratory.infections', 'Cardiovascular.diseases', 'Hospitals', 'Temperature', 'Average.years.of.schooling', 'Stroke'] |
| 13 | 10075.90774 | ['HAQ.index', 'Fully.vaccinated', 'NCD', 'GDP.per.capita', 'Trust.the.national.government', 'Chronic.kidney.disease', 'Booster', 'Cancers', 'Upper.respiratory.infections', 'PM2.5', 'Gender.ratio', 'Hospital.beds', 'Mental.disorders'] |
| 18 | 10113.84086 | ['HAQ.index', 'Fully.vaccinated', 'NCD', 'GDP.per.capita', 'Trust.the.national.government', 'Chronic.kidney.disease', 'Booster', 'Cancers', 'Upper.respiratory.infections', 'Hospital.beds', 'Gender.ratio', 'Overweight', 'IHR.score', 'Mental.disorders', 'PM2.5', 'Trust.science', 'Trust.journalists', 'COPD'] |
| 28 | 10129.27596 | ['HAQ.index', 'Fully.vaccinated', 'NCD', 'GDP.per.capita', 'Trust.the.national.government', 'Booster', 'Chronic.kidney.disease', 'Cancers', 'Upper.respiratory.infections', 'Gender.ratio', 'Hospital.beds', 'PM2.5', 'Mental.disorders', 'IHR.score', 'Trust.science', 'Overweight', 'COPD', 'Low.physical.activity', 'Hypertension', 'Diabetes', 'Dietary.risks', 'Population.density', 'Trust.journalists', 'Trees.per.capita', 'Expenditure', 'Tuberculosis', 'Lower.respiratory.infections', 'Cardiovascular.diseases'] |
| 29 | 10137.01383 | ['HAQ.index', 'Fully.vaccinated', 'NCD', 'GDP.per.capita', 'Trust.the.national.government', 'Booster', 'Chronic.kidney.disease', 'Cancers', 'Upper.respiratory.infections', 'Gender.ratio', 'Hospital.beds', 'PM2.5', 'Mental.disorders', 'IHR.score', 'Trust.science', 'Overweight', 'COPD', 'Low.physical.activity', 'Hypertension', 'Diabetes', 'Dietary.risks', 'Population.density', 'Trust.journalists', 'Trees.per.capita', 'Expenditure', 'Tuberculosis', 'Lower.respiratory.infections', 'Cardiovascular.diseases', 'Hospitals'] |
| 31 | 10139.63849 | ['HAQ.index', 'Fully.vaccinated', 'NCD', 'GDP.per.capita', 'Trust.the.national.government', 'Booster', 'Chronic.kidney.disease', 'Cancers', 'Upper.respiratory.infections', 'Gender.ratio', 'Hospital.beds', 'PM2.5', 'Mental.disorders', 'IHR.score', 'Trust.science', 'Overweight', 'COPD', 'Low.physical.activity', 'Hypertension', 'Diabetes', 'Dietary.risks', 'Population.density', 'Trust.journalists', 'Trees.per.capita', 'Expenditure', 'Tuberculosis', 'Lower.respiratory.infections', 'Cardiovascular.diseases', 'Hospitals', 'Temperature', 'Average.years.of.schooling'] |
| 30 | 10150.14776 | ['HAQ.index', 'Fully.vaccinated', 'NCD', 'GDP.per.capita', 'Trust.the.national.government', 'Booster', 'Chronic.kidney.disease', 'Cancers', 'Upper.respiratory.infections', 'Gender.ratio', 'Hospital.beds', 'PM2.5', 'Mental.disorders', 'IHR.score', 'Trust.science', 'Overweight', 'COPD', 'Low.physical.activity', 'Hypertension', 'Diabetes', 'Dietary.risks', 'Population.density', 'Trust.journalists', 'Trees.per.capita', 'Expenditure', 'Tuberculosis', 'Lower.respiratory.infections', 'Cardiovascular.diseases', 'Hospitals', 'Temperature'] |
| 27 | 10233.84922 | ['HAQ.index', 'Fully.vaccinated', 'NCD', 'GDP.per.capita', 'Trust.the.national.government', 'Booster', 'Chronic.kidney.disease', 'Cancers', 'Upper.respiratory.infections', 'Gender.ratio', 'Hospital.beds', 'PM2.5', 'Mental.disorders', 'IHR.score', 'Trust.science', 'Overweight', 'COPD', 'Low.physical.activity', 'Hypertension', 'Diabetes', 'Dietary.risks', 'Population.density', 'Trust.journalists', 'Trees.per.capita', 'Expenditure', 'Tuberculosis', 'Lower.respiratory.infections'] |
| 16 | 10253.80976 | ['HAQ.index', 'Fully.vaccinated', 'NCD', 'GDP.per.capita', 'Trust.the.national.government', 'Booster', 'Chronic.kidney.disease', 'Upper.respiratory.infections', 'Cancers', 'PM2.5', 'Mental.disorders', 'Trust.journalists', 'Hospital.beds', 'Trust.science', 'Gender.ratio', 'Overweight'] |
| 15 | 10318.19291 | ['HAQ.index', 'Fully.vaccinated', 'NCD', 'GDP.per.capita', 'Trust.the.national.government', 'Chronic.kidney.disease', 'Booster', 'Upper.respiratory.infections', 'Cancers', 'PM2.5', 'Hospital.beds', 'Trust.journalists', 'Mental.disorders', 'Trust.science', 'Gender.ratio'] |
| 14 | 10323.7869 | ['HAQ.index', 'Fully.vaccinated', 'NCD', 'GDP.per.capita', 'Trust.the.national.government', 'Booster', 'Chronic.kidney.disease', 'Upper.respiratory.infections', 'Cancers', 'PM2.5', 'Mental.disorders', 'Hospital.beds', 'Trust.journalists', 'Gender.ratio'] |
| 33 | 10355.6403 | ['HAQ.index', 'GDP.per.capita', 'Fully.vaccinated', 'Trust.the.national.government', 'NCD', 'Booster', 'Gender.ratio', 'Chronic.kidney.disease', 'Overweight', 'COPD', 'Upper.respiratory.infections', 'Cancers', 'Hospital.beds', 'PM2.5', 'Mental.disorders', 'Temperature', 'Trust.science', 'IHR.score', 'Trees.per.capita', 'Low.physical.activity', 'Hypertension', 'Trust.journalists', 'Diabetes', 'Dietary.risks', 'Population.density', 'Stroke', 'Expenditure', 'Average.years.of.schooling', 'Tuberculosis', 'Lower.respiratory.infections', 'Hospitals', 'Cardiovascular.diseases', 'HIV'] |
| 7 | 10377.17596 | ['HAQ.index', 'Fully.vaccinated', 'NCD', 'GDP.per.capita', 'Trust.the.national.government', 'Chronic.kidney.disease', 'Booster'] |
| 12 | 10421.48062 | ['HAQ.index', 'Fully.vaccinated', 'NCD', 'GDP.per.capita', 'Trust.the.national.government', 'Chronic.kidney.disease', 'Booster', 'Upper.respiratory.infections', 'Cancers', 'PM2.5', 'Gender.ratio', 'Mental.disorders'] |
| 6 | 10430.72771 | ['HAQ.index', 'Fully.vaccinated', 'NCD', 'GDP.per.capita', 'Trust.the.national.government', 'Booster'] |
| 11 | 10436.88228 | ['HAQ.index', 'Fully.vaccinated', 'NCD', 'GDP.per.capita', 'Trust.the.national.government', 'Booster', 'Chronic.kidney.disease', 'Upper.respiratory.infections', 'Cancers', 'PM2.5', 'Mental.disorders'] |
| 9 | 10636.30803 | ['HAQ.index', 'Fully.vaccinated', 'NCD', 'GDP.per.capita', 'Trust.the.national.government', 'Chronic.kidney.disease', 'Upper.respiratory.infections', 'PM2.5', 'Booster'] |
| 10 | 10665.43482 | ['HAQ.index', 'Fully.vaccinated', 'NCD', 'GDP.per.capita', 'Trust.the.national.government', 'Chronic.kidney.disease', 'Upper.respiratory.infections', 'Booster', 'PM2.5', 'Cancers'] |
| 8 | 10677.21936 | ['HAQ.index', 'Fully.vaccinated', 'NCD', 'GDP.per.capita', 'Trust.the.national.government', 'Booster', 'Upper.respiratory.infections', 'Chronic.kidney.disease'] |
| 5 | 10848.4912 | ['HAQ.index', 'Fully.vaccinated', 'GDP.per.capita', 'NCD', 'Trust.the.national.government'] |

1. Feature selection of Omicron period model

| **Number of variable** | **RMSE (per 1,000,000 population)** | **Variables** |
| --- | --- | --- |
| 11 | 8895.809837 | ['GDP.per.capita', 'HAQ.index', 'Fully.vaccinated', 'Booster', 'PM2.5', 'NCD', 'Dietary.risks', 'Lower.respiratory.infections', 'Low.physical.activity', 'Trust.science', 'Smoking'] |
| 33 | 8976.852136 | ['HAQ.index', 'GDP.per.capita', 'Fully.vaccinated', 'Booster', 'NCD', 'PM2.5', 'Dietary.risks', 'Smoking', 'Expenditure', 'Cancers', 'Average.years.of.schooling', 'Overweight', 'Lower.respiratory.infections', 'Stroke', 'Hospitals', 'COPD', 'Trust.science', 'Low.physical.activity', 'Chronic.kidney.disease', 'Upper.respiratory.infections', 'Trust.the.national.government', 'Hospital.beds', 'IHR.score', 'Gender.ratio', 'Cardiovascular.diseases', 'Hypertension', 'Mental.disorders', 'Temperature', 'Trees.per.capita', 'Population.density', 'HIV', 'Tuberculosis', 'Trust.journalists'] |
| 24 | 9016.463426 | ['HAQ.index', 'GDP.per.capita', 'Fully.vaccinated', 'Booster', 'NCD', 'Dietary.risks', 'PM2.5', 'Expenditure', 'Smoking', 'Cancers', 'Average.years.of.schooling', 'Stroke', 'Lower.respiratory.infections', 'Trust.science', 'Hospitals', 'COPD', 'Low.physical.activity', 'Chronic.kidney.disease', 'Upper.respiratory.infections', 'Trust.the.national.government', 'IHR.score', 'Hypertension', 'Mental.disorders', 'Temperature'] |
| 18 | 9017.383252 | ['HAQ.index', 'GDP.per.capita', 'Fully.vaccinated', 'Booster', 'PM2.5', 'Dietary.risks', 'NCD', 'Lower.respiratory.infections', 'Smoking', 'Low.physical.activity', 'Average.years.of.schooling', 'Cancers', 'Trust.science', 'IHR.score', 'Stroke', 'COPD', 'Upper.respiratory.infections', 'Chronic.kidney.disease'] |
| 31 | 9030.694549 | ['HAQ.index', 'GDP.per.capita', 'Fully.vaccinated', 'Booster', 'NCD', 'Dietary.risks', 'PM2.5', 'Expenditure', 'Smoking', 'Cancers', 'Average.years.of.schooling', 'Stroke', 'Lower.respiratory.infections', 'Trust.science', 'Hospitals', 'COPD', 'Low.physical.activity', 'Chronic.kidney.disease', 'Upper.respiratory.infections', 'Trust.the.national.government', 'IHR.score', 'Hypertension', 'Mental.disorders', 'Temperature', 'Overweight', 'Population.density', 'Tuberculosis', 'HIV', 'Gender.ratio', 'Trees.per.capita', 'Cardiovascular.diseases'] |
| 10 | 9044.584422 | ['GDP.per.capita', 'HAQ.index', 'Fully.vaccinated', 'Booster', 'PM2.5', 'NCD', 'Dietary.risks', 'Lower.respiratory.infections', 'Trust.science', 'Low.physical.activity'] |
| 19 | 9048.78819 | ['GDP.per.capita', 'HAQ.index', 'Fully.vaccinated', 'Booster', 'NCD', 'PM2.5', 'Lower.respiratory.infections', 'Dietary.risks', 'Smoking', 'Stroke', 'Cancers', 'Trust.science', 'Average.years.of.schooling', 'Low.physical.activity', 'Chronic.kidney.disease', 'Upper.respiratory.infections', 'Hospitals', 'IHR.score', 'COPD'] |
| 30 | 9052.184173 | ['HAQ.index', 'GDP.per.capita', 'Fully.vaccinated', 'Booster', 'NCD', 'Dietary.risks', 'PM2.5', 'Expenditure', 'Smoking', 'Cancers', 'Average.years.of.schooling', 'Stroke', 'Lower.respiratory.infections', 'Trust.science', 'Hospitals', 'COPD', 'Low.physical.activity', 'Chronic.kidney.disease', 'Upper.respiratory.infections', 'Trust.the.national.government', 'IHR.score', 'Hypertension', 'Mental.disorders', 'Temperature', 'Overweight', 'Population.density', 'Tuberculosis', 'HIV', 'Gender.ratio', 'Trees.per.capita'] |
| 23 | 9057.251786 | ['HAQ.index', 'GDP.per.capita', 'Fully.vaccinated', 'Booster', 'NCD', 'Dietary.risks', 'PM2.5', 'Expenditure', 'Smoking', 'Cancers', 'Average.years.of.schooling', 'Stroke', 'Lower.respiratory.infections', 'Trust.science', 'Hospitals', 'COPD', 'Low.physical.activity', 'Chronic.kidney.disease', 'Upper.respiratory.infections', 'Trust.the.national.government', 'IHR.score', 'Hypertension', 'Mental.disorders'] |
| 8 | 9068.239135 | ['GDP.per.capita', 'HAQ.index', 'Fully.vaccinated', 'Booster', 'PM2.5', 'NCD', 'Dietary.risks', 'Lower.respiratory.infections'] |
| 32 | 9070.606633 | ['HAQ.index', 'GDP.per.capita', 'Fully.vaccinated', 'Booster', 'NCD', 'Dietary.risks', 'PM2.5', 'Expenditure', 'Smoking', 'Cancers', 'Average.years.of.schooling', 'Lower.respiratory.infections', 'Stroke', 'Trust.science', 'Hospitals', 'COPD', 'Low.physical.activity', 'Chronic.kidney.disease', 'Overweight', 'Upper.respiratory.infections', 'Trust.the.national.government', 'IHR.score', 'Hypertension', 'Mental.disorders', 'Temperature', 'Population.density', 'Tuberculosis', 'HIV', 'Gender.ratio', 'Trees.per.capita', 'Cardiovascular.diseases', 'Hospital.beds'] |
| 9 | 9085.8747 | ['HAQ.index', 'GDP.per.capita', 'Fully.vaccinated', 'Booster', 'PM2.5', 'Dietary.risks', 'NCD', 'Lower.respiratory.infections', 'Trust.science'] |
| 28 | 9090.305013 | ['HAQ.index', 'GDP.per.capita', 'Fully.vaccinated', 'Booster', 'NCD', 'Dietary.risks', 'PM2.5', 'Expenditure', 'Smoking', 'Cancers', 'Average.years.of.schooling', 'Stroke', 'Lower.respiratory.infections', 'Trust.science', 'Hospitals', 'COPD', 'Low.physical.activity', 'Chronic.kidney.disease', 'Upper.respiratory.infections', 'Trust.the.national.government', 'IHR.score', 'Hypertension', 'Mental.disorders', 'Temperature', 'Overweight', 'Population.density', 'Tuberculosis', 'HIV'] |
| 25 | 9093.3489 | ['HAQ.index', 'GDP.per.capita', 'Fully.vaccinated', 'Booster', 'NCD', 'Dietary.risks', 'PM2.5', 'Expenditure', 'Smoking', 'Cancers', 'Average.years.of.schooling', 'Stroke', 'Lower.respiratory.infections', 'Trust.science', 'Hospitals', 'COPD', 'Low.physical.activity', 'Chronic.kidney.disease', 'Upper.respiratory.infections', 'Trust.the.national.government', 'IHR.score', 'Hypertension', 'Mental.disorders', 'Temperature', 'Overweight'] |
| 26 | 9121.064234 | ['HAQ.index', 'GDP.per.capita', 'Fully.vaccinated', 'Booster', 'NCD', 'Dietary.risks', 'PM2.5', 'Expenditure', 'Smoking', 'Cancers', 'Average.years.of.schooling', 'Stroke', 'Lower.respiratory.infections', 'Trust.science', 'Hospitals', 'COPD', 'Low.physical.activity', 'Chronic.kidney.disease', 'Upper.respiratory.infections', 'Trust.the.national.government', 'IHR.score', 'Hypertension', 'Mental.disorders', 'Temperature', 'Overweight', 'Population.density'] |
| 27 | 9134.999737 | ['HAQ.index', 'GDP.per.capita', 'Fully.vaccinated', 'Booster', 'NCD', 'Dietary.risks', 'PM2.5', 'Expenditure', 'Smoking', 'Cancers', 'Average.years.of.schooling', 'Stroke', 'Lower.respiratory.infections', 'Trust.science', 'Hospitals', 'COPD', 'Low.physical.activity', 'Chronic.kidney.disease', 'Upper.respiratory.infections', 'Trust.the.national.government', 'IHR.score', 'Hypertension', 'Mental.disorders', 'Temperature', 'Overweight', 'Population.density', 'Tuberculosis'] |
| 16 | 9135.06167 | ['GDP.per.capita', 'HAQ.index', 'Fully.vaccinated', 'Booster', 'PM2.5', 'Dietary.risks', 'Lower.respiratory.infections', 'NCD', 'Smoking', 'Low.physical.activity', 'Cancers', 'IHR.score', 'Trust.science', 'Stroke', 'Upper.respiratory.infections', 'Average.years.of.schooling'] |
| 6 | 9152.241736 | ['GDP.per.capita', 'HAQ.index', 'Fully.vaccinated', 'Booster', 'PM2.5', 'Dietary.risks'] |
| 17 | 9158.162618 | ['HAQ.index', 'GDP.per.capita', 'Fully.vaccinated', 'Booster', 'PM2.5', 'Dietary.risks', 'NCD', 'Lower.respiratory.infections', 'Smoking', 'Low.physical.activity', 'Cancers', 'Trust.science', 'IHR.score', 'Stroke', 'COPD', 'Average.years.of.schooling', 'Upper.respiratory.infections'] |
| 29 | 9161.252075 | ['HAQ.index', 'GDP.per.capita', 'Fully.vaccinated', 'Booster', 'NCD', 'Dietary.risks', 'PM2.5', 'Expenditure', 'Smoking', 'Cancers', 'Average.years.of.schooling', 'Stroke', 'Lower.respiratory.infections', 'Trust.science', 'Hospitals', 'COPD', 'Low.physical.activity', 'Chronic.kidney.disease', 'Upper.respiratory.infections', 'Trust.the.national.government', 'IHR.score', 'Hypertension', 'Mental.disorders', 'Temperature', 'Overweight', 'Population.density', 'Tuberculosis', 'HIV', 'Gender.ratio'] |
| 14 | 9162.088518 | ['GDP.per.capita', 'HAQ.index', 'Fully.vaccinated', 'Booster', 'PM2.5', 'NCD', 'Dietary.risks', 'Lower.respiratory.infections', 'Low.physical.activity', 'Smoking', 'IHR.score', 'Cancers', 'Stroke', 'Trust.science'] |
| 22 | 9164.496503 | ['GDP.per.capita', 'HAQ.index', 'Fully.vaccinated', 'Booster', 'NCD', 'PM2.5', 'Dietary.risks', 'Lower.respiratory.infections', 'Smoking', 'Stroke', 'Cancers', 'Trust.science', 'Average.years.of.schooling', 'Low.physical.activity', 'Chronic.kidney.disease', 'Upper.respiratory.infections', 'Hospitals', 'IHR.score', 'COPD', 'Mental.disorders', 'Trust.the.national.government', 'Expenditure'] |
| 20 | 9176.62474 | ['GDP.per.capita', 'HAQ.index', 'Fully.vaccinated', 'Booster', 'NCD', 'PM2.5', 'Dietary.risks', 'Lower.respiratory.infections', 'Smoking', 'Stroke', 'Cancers', 'Trust.science', 'Average.years.of.schooling', 'Low.physical.activity', 'Chronic.kidney.disease', 'Upper.respiratory.infections', 'Hospitals', 'IHR.score', 'COPD', 'Mental.disorders'] |
| 21 | 9178.022244 | ['GDP.per.capita', 'HAQ.index', 'Fully.vaccinated', 'Booster', 'NCD', 'PM2.5', 'Lower.respiratory.infections', 'Dietary.risks', 'Smoking', 'Stroke', 'Cancers', 'Trust.science', 'Average.years.of.schooling', 'Low.physical.activity', 'Chronic.kidney.disease', 'Upper.respiratory.infections', 'Hospitals', 'IHR.score', 'COPD', 'Mental.disorders', 'Trust.the.national.government'] |
| 12 | 9186.79439 | ['GDP.per.capita', 'HAQ.index', 'Fully.vaccinated', 'Booster', 'PM2.5', 'NCD', 'Dietary.risks', 'Lower.respiratory.infections', 'Smoking', 'Low.physical.activity', 'Trust.science', 'Cancers'] |
| 7 | 9294.056076 | ['GDP.per.capita', 'HAQ.index', 'Fully.vaccinated', 'Booster', 'PM2.5', 'Dietary.risks', 'NCD'] |
| 15 | 9326.919758 | ['HAQ.index', 'GDP.per.capita', 'Fully.vaccinated', 'Booster', 'PM2.5', 'Dietary.risks', 'Lower.respiratory.infections', 'NCD', 'Smoking', 'Low.physical.activity', 'Cancers', 'Trust.science', 'Stroke', 'IHR.score', 'Upper.respiratory.infections'] |
| 13 | 9400.810116 | ['GDP.per.capita', 'HAQ.index', 'Fully.vaccinated', 'Booster', 'PM2.5', 'Lower.respiratory.infections', 'Dietary.risks', 'NCD', 'Low.physical.activity', 'Smoking', 'Cancers', 'IHR.score', 'Trust.science'] |
| 5 | 9557.456523 | ['GDP.per.capita', 'HAQ.index', 'Fully.vaccinated', 'Booster', 'Dietary.risks'] |

**Supplementary table 3. Table of SHAP values by factor for each country**

| Country | GDP per capita | HAQ index | Fully vaccinated | Booster | PM2.5 | NCD | Dietary risks | Lower respiratory infections | Low physical activity | Trust science | Smoking |
| --- | --- | --- | --- | --- | --- | --- | --- | --- | --- | --- | --- |
| Indonesia | 0.0741 | 0.0378 | -1.0030 | 0.1143 | -0.0611 | 0.0165 | 0.0309 | 0.0053 | -0.0902 | 0.0271 | -0.0008 |
| Nigeria | -0.2325 | 0.0677 | -2.7713 | 0.2176 | 0.0427 | 0.0115 | 0.0262 | 0.0105 | 0.0083 | 0.0060 | 0.0004 |
| Somalia | 0.0902 | 0.0824 | -1.0066 | 0.1294 | 0.0436 | 0.0115 | 0.0165 | -0.0421 | -0.0214 | -0.0011 | -0.0056 |
| Argentina | -0.8852 | 0.0341 | -0.1617 | -0.2278 | -0.1006 | -0.0611 | 0.1157 | 0.0105 | -0.0220 | -0.0126 | -0.0545 |
| Finland | -0.9477 | -0.4304 | -0.0645 | -0.3588 | -0.1258 | 0.0296 | 0.2069 | -0.0296 | -0.0235 | -0.0109 | 0.1294 |
| Greece | -1.1005 | -0.1254 | -0.0814 | -0.4204 | -0.1028 | 0.0021 | 0.1273 | -0.0285 | 0.0133 | -0.0002 | -0.0371 |
| New Zealand | -0.9243 | -0.3928 | -0.1433 | -0.2509 | -0.1618 | -0.4011 | 0.0257 | -0.0190 | -0.0322 | -0.0101 | -0.0818 |
| Slovenia | -1.0988 | -0.3992 | -0.4027 | -0.2646 | -0.0807 | 0.0020 | 0.1376 | -0.0891 | -0.0314 | 0.0006 | -0.0526 |
| USA | -0.8792 | 0.1101 | -0.3122 | -0.2120 | -0.0762 | 0.0375 | 0.1401 | 0.0397 | 0.0254 | 0.0030 | -0.0439 |
| Bulgaria | 0.6005 | 0.0736 | -0.7658 | 0.0389 | 0.0104 | 0.0147 | 0.0172 | 0.0496 | -0.0873 | -0.0002 | 0.0010 |
| Colombia | 0.6468 | 0.0527 | 0.1110 | -0.0064 | -0.0042 | 0.0199 | 0.0467 | 0.0193 | -0.0165 | 0.0315 | 0.0273 |
| Jamaica | 0.6774 | 0.0629 | 0.1880 | 0.1095 | -0.0140 | 0.0172 | 0.0370 | 0.0549 | -0.0892 | -0.0002 | 0.0004 |
| Peru | 0.5286 | 0.0809 | -0.1247 | -0.2171 | 0.0479 | 0.0167 | -0.0822 | 0.0017 | -0.0236 | 0.0297 | 0.0352 |
| Philippines | 0.4863 | 0.0567 | 0.1262 | -0.5270 | 0.0182 | 0.0123 | 0.0246 | -0.0220 | -0.0206 | -0.0007 | 0.0017 |
| Togo | 0.4082 | 0.0593 | -0.7915 | 0.1852 | 0.0232 | 0.0115 | 0.0142 | -0.0050 | 0.0098 | -0.0002 | -0.0062 |
| Chile | -1.0837 | 0.1622 | -0.1267 | -0.6036 | 0.1253 | -0.0029 | 0.0720 | -0.0069 | -0.0081 | 0.0224 | 0.0576 |
| Czechia | -1.2175 | 0.0850 | -0.1777 | -0.4983 | -0.1393 | 0.0023 | 0.0239 | -0.0891 | -0.1370 | 0.0030 | -0.0572 |
| Slovakia | -0.9881 | 0.1260 | -0.6601 | -0.2859 | 0.0551 | 0.0343 | -0.0177 | 0.0356 | -0.0874 | 0.0030 | -0.0572 |
| Australia | -0.9807 | -0.4887 | -0.2115 | -0.0777 | -0.1200 | -0.0753 | -0.2833 | -0.0179 | 0.1183 | -0.0100 | 0.0909 |
| Austria | -1.2862 | -0.7642 | -0.3702 | -0.6929 | -0.0184 | 0.0073 | -0.1300 | -0.0863 | 0.0869 | -0.0017 | -0.0457 |
| Belgium | -1.1407 | -0.6813 | -0.2994 | -0.3356 | -0.0263 | -0.0379 | -0.0931 | -0.0234 | 0.1299 | 0.0004 | -0.0421 |
| Germany | -1.2399 | -0.8230 | -0.2557 | -0.6400 | -0.1071 | 0.0073 | -0.0619 | -0.0303 | 0.0671 | -0.0017 | -0.0457 |
| Italy | -1.2860 | -0.6629 | -0.0886 | -0.5464 | -0.0221 | 0.0415 | -0.0998 | -0.0225 | 0.0985 | 0.0004 | -0.0421 |
| Spain | -1.2403 | -0.5896 | -0.0705 | -0.2759 | -0.1379 | -0.0555 | -0.2251 | -0.0342 | 0.1214 | 0.0031 | -0.0421 |
| Portugal | -1.5671 | -0.0146 | -0.2865 | -0.3056 | -0.1708 | 0.0175 | -0.3144 | -0.0289 | -0.6989 | -0.0100 | -0.0641 |
| Sweden | -1.1972 | -0.7839 | -0.0472 | 0.0224 | -0.0994 | 0.0368 | -0.4597 | -0.0349 | -0.2471 | -0.0123 | -0.0348 |
| Isreal | -0.9036 | 0.1002 | -0.6504 | -0.1512 | 0.1986 | 0.0314 | -0.2023 | -0.0505 | -0.0106 | 0.0004 | 0.1126 |
| South Korea | -1.0761 | 0.0352 | -0.3638 | -0.9930 | 0.0848 | -0.0531 | 0.0172 | -0.0148 | 0.0262 | -0.0179 | -0.0059 |
| Switzerland | -1.2968 | -0.9618 | -0.2821 | -0.3281 | -0.1315 | -0.0502 | -0.2488 | -0.0893 | -0.2903 | 0.0031 | -0.0490 |
| Denmark | -1.1590 | -0.8596 | -0.0765 | -0.4455 | -0.1411 | -0.0484 | -0.2533 | -0.0344 | -0.1614 | -0.0127 | -0.0440 |
| France | -1.2744 | -0.8960 | -0.0514 | -0.4198 | -0.1339 | -0.0502 | -0.1731 | -0.0330 | -0.1946 | -0.0074 | -0.0421 |
| Japan | -1.2272 | -0.6966 | -0.1060 | -0.1986 | -0.1356 | -0.0906 | -0.5482 | -0.0234 | -0.2547 | -0.0114 | -0.0976 |

**Supplementary table 4. CFR reduction rate after increasing booster vaccination rates**

| **Country** | **Increase in booster vaccination rates** | **Reduction rate of CFR** |
| --- | --- | --- |
| Nigeria | 1% | 2% |
| Togo | 1% | 3% |
| Isreal | 1% | 9% |
| New Zealand | 1% | 14% |
| Spain | 1% | 3% |
| France | 1% | 4% |
| Greece | 1% | 24% |
| Japan | 1% | 22% |
| Indonesia | 2% | 12% |
| Peru | 2% | 1% |
| Sweden | 2% | 9% |
| Finland | 2% | 9% |
| Colombia | 3% | 9% |
| Jamaica | 3% | 15% |
| Slovenia | 3% | 14% |
| Philippines | 4% | 50% |
| USA | 4% | 14% |
| Argentina | 5% | 14% |
| South Korea | 7% | 22% |
| Switzerland | 7% | 3% |
| Bulgaria | 9% | 1% |
| Czechia | 12% | 9% |
| Australia | 14% | 6% |
| Portugal | 17% | 9% |

**Supplementary figure 1. Correlation between covariates**


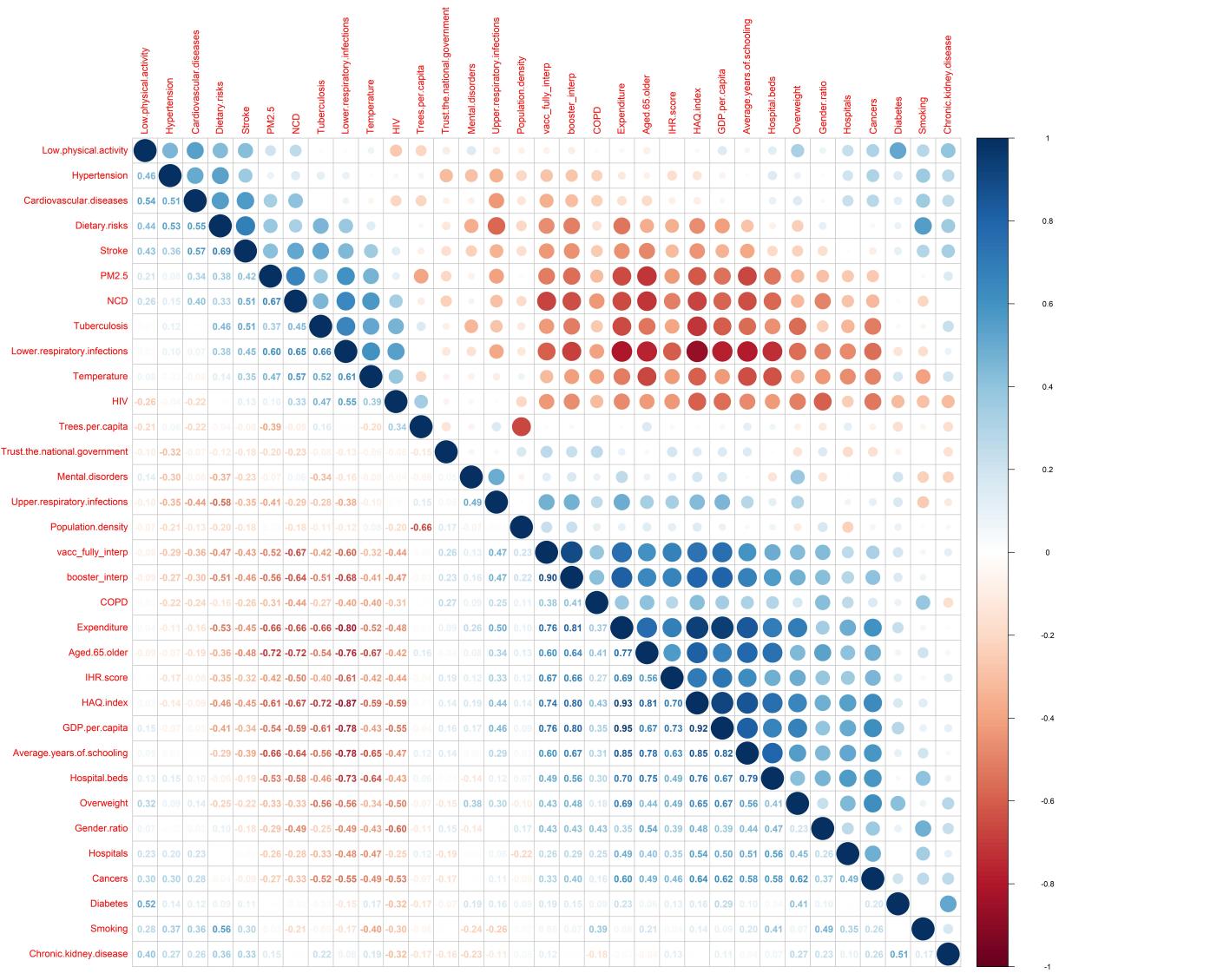


**Supplementary figure 2. The percentage increase in booster vaccination rates when the CFR of each country shows a decrease. “*” is countries with higher age-adjusted CFRs than crude CFRs.**


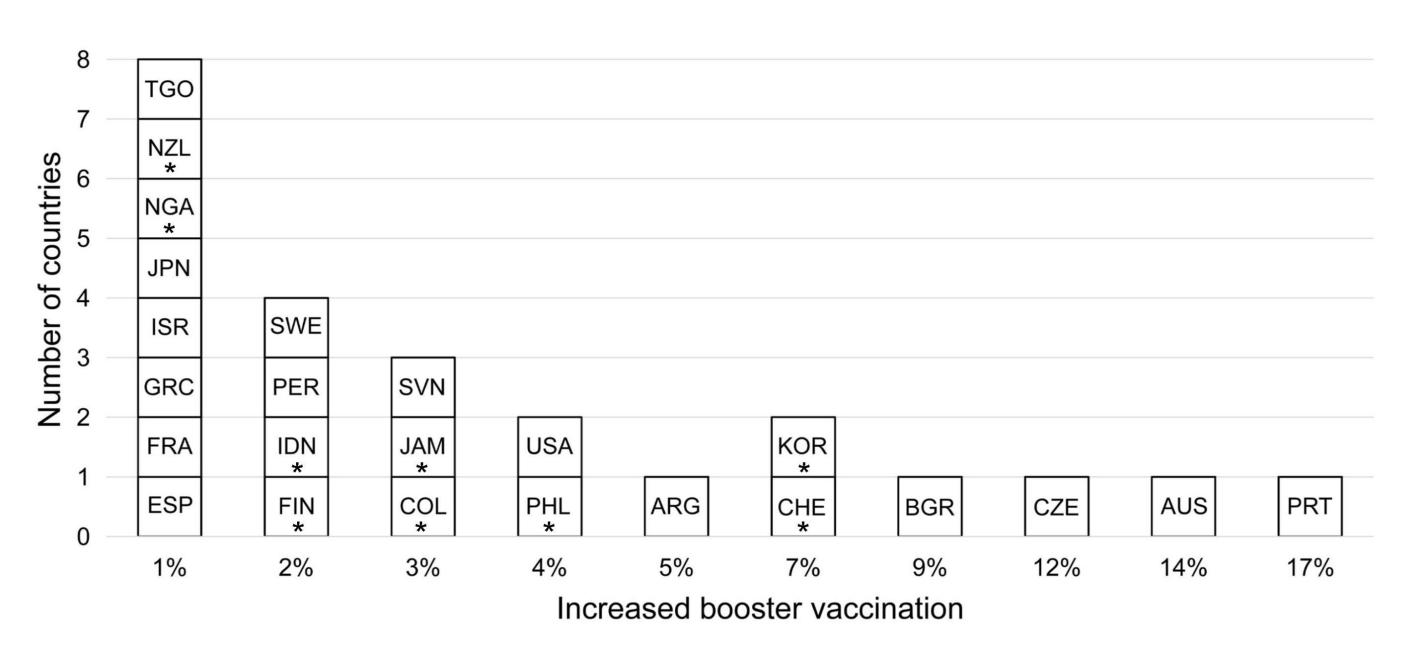

Supplement: Supplementary file 1 [file Data_Sheet_1.docx]
